# Super-resolution neural networks improve the spatiotemporal resolution of adaptive MRI-guided radiation therapy

**DOI:** 10.1038/s43856-024-00489-9

**Published:** 2024-04-04

**Authors:** James Grover, Paul Liu, Bin Dong, Shanshan Shan, Brendan Whelan, Paul Keall, David E. J. Waddington

**Affiliations:** 1 Image X Institute, Sydney School of Health Sciences, Faculty of Medicine and Health, The University of Sydney, Sydney, NSW Australia; 2 Department of Medical Physics, Ingham Institute for Applied Medical Research, Sydney, NSW Australia; 3 State Key Laboratory of Radiation Medicine and Protection, School for Radiological and Interdisciplinary Sciences (RAD-X), Collaborative Innovation Center of Radiation Medicine of Jiangsu Higher Education Institutions, Soochow University, Suzhou, Jiangsu China

**Keywords:** Radiotherapy, Magnetic resonance imaging

## Abstract

**Background:**

Magnetic resonance imaging (MRI) offers superb non-invasive, soft tissue imaging of the human body. However, extensive data sampling requirements severely restrict the spatiotemporal resolution achievable with MRI. This limits the modality’s utility in real-time guidance applications, particularly for the rapidly growing MRI-guided radiation therapy approach to cancer treatment. Recent advances in artificial intelligence (AI) could reduce the trade-off between the spatial and the temporal resolution of MRI, thus increasing the clinical utility of the imaging modality.

**Methods:**

We trained deep learning-based super-resolution neural networks to increase the spatial resolution of real-time MRI. We developed a framework to integrate neural networks directly onto a 1.0 T MRI-linac enabling real-time super-resolution imaging. We integrated this framework with the targeting system of the MRI-linac to demonstrate real-time beam adaptation with super-resolution-based imaging. We tested the integrated system using large publicly available datasets, healthy volunteer imaging, phantom imaging, and beam tracking experiments using bicubic interpolation as a baseline comparison.

**Results:**

Deep learning-based super-resolution increases the spatial resolution of real-time MRI across a variety of experiments, offering measured performance benefits compared to bicubic interpolation. The temporal resolution is not compromised as measured by a real-time adaptation latency experiment. These two effects, an increase in the spatial resolution with a negligible decrease in the temporal resolution, leads to a net increase in the spatiotemporal resolution.

**Conclusions:**

Deployed super-resolution neural networks can increase the spatiotemporal resolution of real-time MRI. This has applications to domains such as MRI-guided radiation therapy and interventional procedures.

## Plain Language Summary

Magnetic resonance imaging (MRI) is a medical imaging modality that is used to image organs such as the brain, lungs, and liver as well as diseases such as cancer. MRI scans taken at high resolution are of overly long duration. This time constraint limits the accuracy of MRI-guided cancer radiation therapy, where imaging must be fast to adapt treatment to tumour motion. Here, we deployed artificial intelligence (AI) models to achieve fast and high detail MRI. We additionally validated our AI models across various scenarios. These AI-based models could potentially enable people with cancer to be treated with higher accuracy and precision.

## Abstract (web summary)

Grover et al. describe the training, deployment, and evaluation of real-time super-resolution imaging for MRI-guided radiation therapy. Volunteer, phantom, and simulation experiments demonstrate that super-resolution can increase the spatiotemporal resolution of real-time MRI guidance.

## Introduction

Magnetic resonance imaging (MRI) is a non-ionising and non-invasive imaging modality used in a broad range of medical applications. Human anatomy can be visualised in unprecedented detail owing to the inherent imaging physics behind magnetic resonance. Traditionally used to diagnose disease, advances in real-time MRI have enabled new applications in real-time treatment guidance. Real-time MRI has a variety of medical applications including cardiac, upper airway, and musculoskeletal imaging as well as MRI-guided procedures including cardiac radioablation and radiation therapy^1–3^. For real-time treatment guidance a challenge remains: MRI is a slow imaging modality^4^.

Real-time MRI is traditionally governed by the trade-off interplay between spatial resolution, temporal resolution, signal-to-noise ratio, artifacts, reconstruction latency, and modelling assumptions^1^. The main components in MR imaging time are the acquisition time and the image reconstruction time^5^. MRI acquisition refers to the application of magnetic gradients, spin relaxation, and the encoding of the spatial frequency domain (k-space). Image reconstruction refers to the process of converting k-space into the image domain and associated processing. Decreases in the acquisition time have been enabled through techniques such as multi-coil imaging, non-Cartesian k-space encoding, and under-sampling k-space^1,6–9^. Clinical benefits include reduced patient time in scanner, reduced motion blur, and increased framerate (for real-time MRI). However, many of these techniques require additional processing that increase the image reconstruction time delaying the availability of images for treatment guidance.

An application of real-time MRI for treatment guidance is the treatment of cancer through radiation therapy. High doses of ionising radiation are delivered with the intent to control the tumour whilst sparing surrounding healthy tissue from radiation-induced toxicities. A treatment device known as an MRI linear accelerator (MRI-linac) is the synergy of MRI with radiation therapy to deliver MRI guided radiation therapy (MRIgRT)^10^. There is emerging evidence that MRIgRT offers improved dose coverage and reduced radiation-induced toxicities compared to conventional radiation therapy treatments^11–13^. Adaptation of radiation specific to the changing anatomy of the patient encompasses longer term changes (e.g., patient weight change and tumour response) and real-time changes (e.g., tumour motion due to patients’ respiration)^14–16^. Integrating real-time adaptive MRIgRT poses unique challenges, in particular, the spatiotemporal limitations observed in real-time MRI^3,4,17^. Real-time adaptive radiation therapy can incorporate the direct targeting of the precise radiation beam to the moving target in a technique called multi-leaf collimator (MLC) tracking or beam targeting^18,19^.

Deep learning has enabled unprecedented image enhancement across the sciences. A type of image enhancement is super-resolution: the process where higher resolution data are generated from a measured lower resolution through up-sampling^20^. Applications of super-resolution vary to overcome traditional barriers such as the diffraction limit in fluorescence microscopy and prohibitive expense present in space hyperspectral imaging^21,22^. Although offline, retrospective studies showing the potential of super-resolution to improve image quality are prevalent, publications reporting the translation of super-resolution onto real world systems are rare, especially in medical imaging^23–25^.

Currently, the balance of spatiotemporal resolution in real-time adaptive MRIgRT is maintained by using large pixel sizes to ensure temporal latencies are at an acceptable level^5,26,27^. Here, we train and test a leading super-resolution network, showing that it increases the resolution of MRI scans with high accuracy when tested on large external datasets. We then prospectively deploy an open-source integration of super-resolution into a real-time adaptive MRIgRT workflow that allows for real-time MR imaging at increased resolution without compromising the temporal resolution. We validated this integration using volunteers for a variety of sequences and anatomical sites to assess the image quality improvement afforded by integrated super-resolution. Finally, we experimentally demonstrate the real-time performance of our super-resolution deployment by measuring the temporal resolution of the integrated system and guidance latencies through a beam tracking experiment on an MRI-Linac. Our integration of super-resolution enables real-time higher-resolution imaging across a broad range of applications in medicine including interventional radiology.

## Methods

**Fig. 1:**
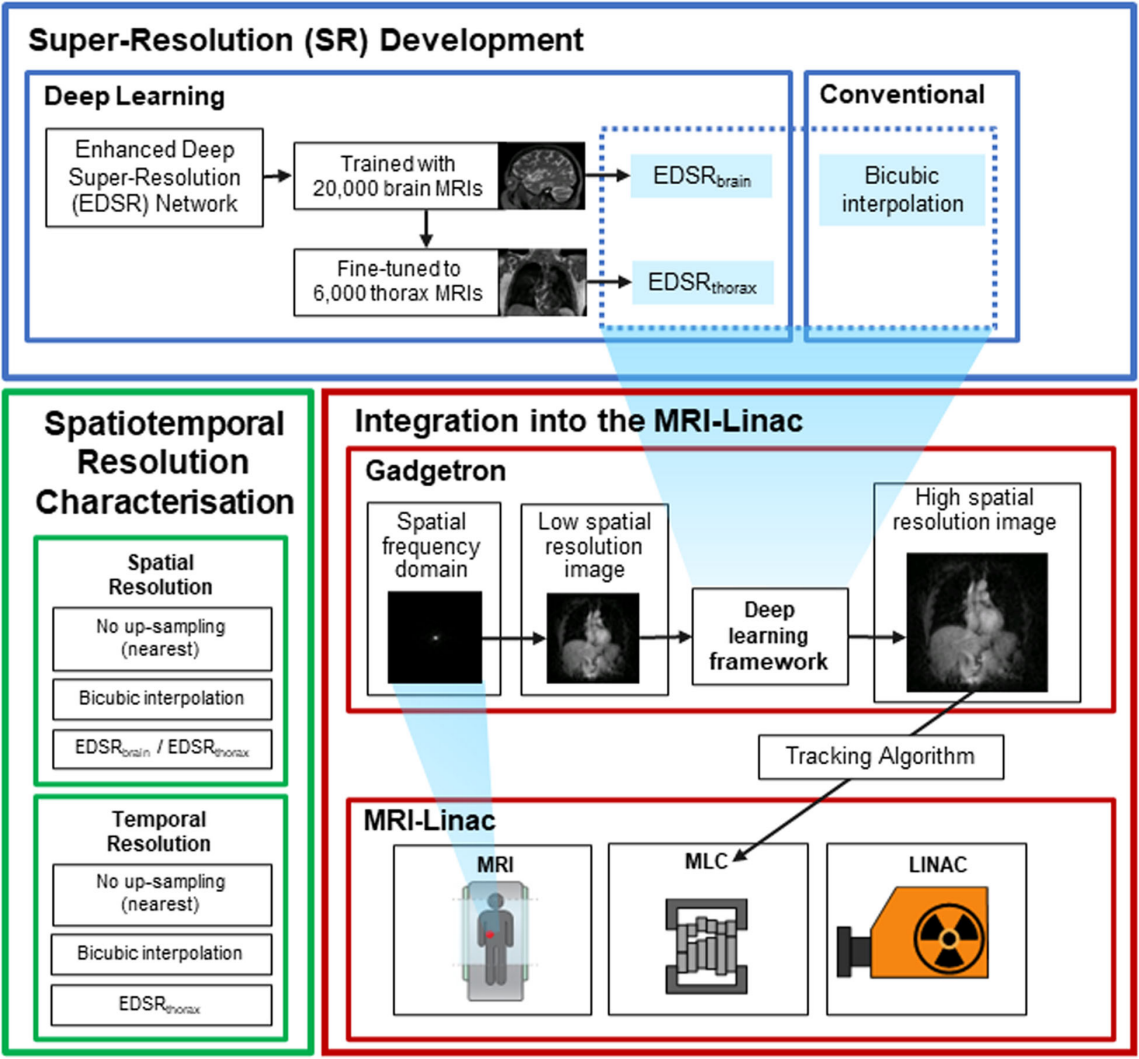
Overview of the methodology. Super-resolution (SR) development incorporated the training of an enhanced deep super-resolution (EDSR) network to brain and thorax MR images to establish a specifically trained brain (EDSR_brain_) and thorax (EDSR_thorax_) models. These deep learning-based and bicubic interpolation (as a conventionally based baseline) techniques were integrated into the MRI-linac through Gadgetron and an in-house framework. Our framework additionally integrated with multi-leaf collimator (MLC) tracking to realise deep learning enhanced real-time treatment guidance. The spatial resolution was assessed comparing super-resolution methods (EDSR_brain_, EDSR_thorax_, and bicubic) with no up-sampling (nearest) to quantify a spatial resolution increase. The temporal resolution was also assessed to measure if super-resolution methods prohibitively increased system latency and their effect on beam tracking.

The methodology of this research is split into three components: super-resolution development, integration into the MRI-linac, and spatiotemporal resolution characterisation. An overview of the methodology is provided in Fig. 1.

### Super-resolution development

The super-resolution methods developed in our integration were 2D single image using a 4× scale factor to up-sample a 64 × 64 MRI matrix size to 256 × 256. To relate this to physical anatomical size, for a commonly used thoracic field-of-view of 400 × 400 mm^2^, the pixel size is transformed from 6.25 × 6.25 mm^2^ to 1.56 × 1.56 mm^2^.

Deep learning-based super-resolution used the enhanced deep residual network for single-image super-resolution (EDSR) architecture^28^. This architecture has a foundational residual block, similar in design to ResNet but with key modifications, such as the removal of batch normalisation^29^. Following residual blocks, an up-sample block comprised of convolution and pixel shuffle layers are used to up-sample image data. The PyTorch framework was used for deep learning development and super-resolution technique compilation described later^30^. The EDSR architecture was modified to accept single channel input and produce a single channel label, as required by grayscale MRI. A further modification was to subtract the mean grayscale (cf. RGB) value of each forward pass. This mean value was subsequently added to the output serving as the method of normalisation for both training and inference.

Dataset selection is of critical importance when training neural networks. In the case of training a large parameter model, it is paramount the dataset resembles the inference application (i.e., MRI) and is large enough as to not overfit. Consequently, the QIN-GBM Treatment Response dataset available on the Cancer Imaging Archive was used to train the EDSR (https://wiki.cancerimagingarchive.net/display/Public/QIN + GBM+Treatment+Response)^31–33^. This dataset contains T1-weighted (pre and post-contrast), T2-weighted, FLAIR, MEMPRAGE along with other advanced sequences for over 50 patients. A script was produced to load specific sequences (T1-weighted pre and post-contrast, T2 weighted, and FLAIR) for training. Choosing multiple sequences was anticipated to improve the generalisability to different sequences on the MRI-linac.

A 2× (corresponding to increasing the spatial resolution by a factor of 2) brain model was trained until convergence of the validation loss. These trained parameters were transferred to a 4× brain model (EDSR_brain_) where additional training was carried out using a higher up-sample ratio for consistency with the original EDSR implementation^28^. EDSR_brain_ was fine-tuned to thorax acquisitions creating a 4× thorax model (EDSR_thorax_) utilising a lung cancer dataset previously described (the AVATAR study)^34,35^. The training pipeline is provided in supplementary method 1 and supplementary fig. 1.

The matrix size varied across different sequences and was not necessarily the target matrix size. For sequences with matrix sizes smaller than the target, these images were zero padded up to the target matrix size. In the case of the matrix size being larger than the target, these images were down-sampled in k-space in a procedure previously described^24^. Down-sampling in k-space is the technique where a high-resolution image has a fast Fourier transform (FFT) applied producing the frequency domain representation of the image. The centre of this frequency domain, corresponding to low spatial frequency information, is cropped to the desired matrix size. This cropped frequency domain then has an inverse FFT applied producing an image with the desired image resolution.

Once the sequences were processed, ensuring (or transforming) each had the correct target matrix size, pre-processing for the neural network training commenced. Labels were randomly cropped, made possible by the EDSR architecture being independent of input matrix size, for further consistency with the literature^24,28,36^. Inputs were generated inline per batch from the cropped labels. To generate corresponding inputs, each label image was down-sampled in k-space by a factor of four to produce a low spatial resolution input.

A by-product of removing the periphery of the frequency domain (when down-sampling in k-space) is that high spatial frequency information is lost. Consequently, edges and other finer structures are diminished. To account for this, an edge-based loss function was developed for training and is outlined in the supplementary method 1 being previously described for single-image super-resolution^36^.

The validation loss and metrics: normalised root mean-square-error (NRMSE), structural similarity (SSIM), and peak signal-to-noise ratio (PSNR) were tracked during training. Parameters were saved for integration on the MRI-linac when the edge based L1 loss was minimised. The optimiser used for training was the Adaptive Momentum Estimation (Adam) algorithm^37^. A one-cycle learning rate scheduler was used during training to modulate the learning rate during training^38^. The maximum learning rate assigned to the learning rate scheduler was 1 × 10^−5^, 5 × 10^−5^, 1 × 10^−6^ for the 2× brain, 4× brain, and 4× thorax models respectively. Two 4× deep learning-based super-resolution models were integrated into the MRI-linac (EDSR_brain_ and EDSR_thorax_).

### Integration into MRI-linac

The Gadgetron image reconstruction framework was employed for online (deployed through Docker for prospective experiments) and offline (for retrospective experiments) reconstruction^39,40^. Gadgetron functions through a sequence of gadgets that ultimately reconstruct MR images from acquisitions. We developed a framework that integrated with Gadgetron. This framework utilised generic and custom Gadgetron gadgets, allowing for end-to-end reconstruction, application of deep learning-based super-resolution or bicubic interpolation, and the sending of reconstructed MR images with associated metadata via transmission control protocol to an in-house multi-leaf collimator (MLC) tracking software^5^.

All methods of super-resolution (deep learning-based and bicubic interpolation) were compiled utilising the PyTorch framework. This compilation allowed for the saving of the network/algorithm architecture and weights into a single file that could be loaded efficiently into memory upon use in Gadgetron.

The Australian MRI-linac was the target system in this study^41^. Details of this MRI-linac system pertinent to this study have been described previously^5^. A 1.0 T split bore magnet (Agilent, UK) with a Magnetom Avanto spectrometer-based control system (Siemens, Germany) is used for MR imaging. A 6 MV Linatron linear accelerator (Varex Imaging, USA) complimented by a 120 leaf Millennium MLC (Varian, USA) is used to deliver a conformal treatment beam.

MLC tracking utilised two different technologies: one calculated a displacement vector of the tracked target and the other optimised leaf positions to this displacement vector. Calculation of the displacement vector was achieved through an in-house software that performed template matching to delineate the target position forming a displacement vector <x, y, z> from the initial target position^5^. This displacement vector is provided to the MLC tracking software to optimize leaf positions based on their current position, maximum leaf velocity and other physical properties of the MLC system. These optimised leaf positions are then sent to the MLC controller to actualise treatment adaptation.

### Spatiotemporal resolution experimentation

**Fig. 2:**
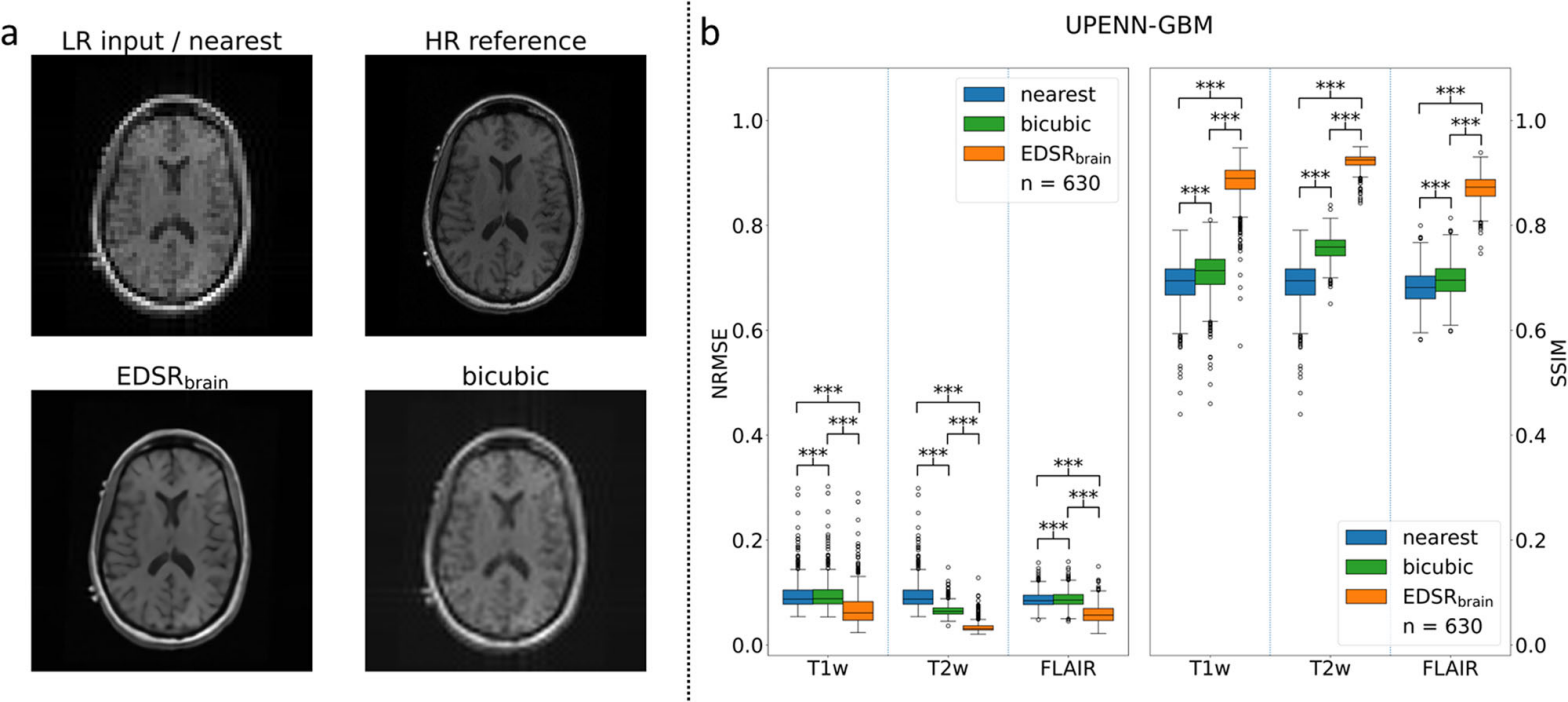
External validation on UPENN-GBM dataset. **a** T1w image from the UPENN-GBM dataset. LR input / nearest is the low spatial resolution input to super-resolution technique (EDSR_brain_ and bicubic) derived from the high spatial resolution label (HR reference). **b** Is a boxplot of quantitative performance measures across the *n* = 630 subjects each containing a T1w, T2w, and FLAIR image. All images scaled between 0 and 1. Asterisks denote statistical significance in a paired *t* test. ns: no statistical significance, *p < 0.05, **p < 0.01, ***p < 0.001. Exact *p* values are given in supplementary data 1. Error bars correspond to the 1.5× interquartile range values. EDSR: enhanced deep super-resolution. NRMSE normalised root mean-square-error, SSIM structural similarity.

External data validation was performed to characterise the generalisability of both super-resolution models (EDSR_brain_ and EDSR_thorax_). The UPENN-GBM dataset consists of T1w, T1w with contrast, T2w, and FLAIR brain images for over 600 subjects (https://wiki.cancerimagingarchive.net/pages/viewpage.action?pageId=70225642)^33,42^. T1w, T2w, and FLAIR sequences were loaded in as a 3D matrix and were either padded with zeros or cropped to ensure a label dimension of [slices, 256, 256]. These label images were down-sampled in k-space on a slice-by-slice basis to input dimensions of [slices, 64, 64]. Both input and label images were min-max scaled between 0 and 4096. These input images were provided to each up-sampling technique (nearest neighbour, bicubic interpolation, EDSR_brain_). The quantitative and qualitative results for this test set are provided in Fig. 2. A similar process was performed for the prostate-diagnosis dataset (https://wiki.cancerimagingarchive.net/display/Public/PROSTATE-DIAGNOSIS)^33,43^. Prior to the generation of the inputs, the labels had any voxel value > 4096 set equal to 4096 as some of the images in the dataset had small regions of extremely high intensity ( > 10,000). These input images were then provided to each up-sampling technique (nearest neighbour, bicubic interpolation, EDSR_thorax_). The quantitative and qualitative results for this test set are provided in supplementary discussion 1 and supplementary fig. 2 as part of a hallucination analysis.

To test the efficacy of our integration of super-resolution to enhance real-time tracking we tested the spatiotemporal resolution by phantom imaging and volunteer imaging. Quantitative analysis was performed using Python and an in-house electronic portal imaging detector (EPID) tracking software using MATLAB (The MathWorks Inc., USA). Acquisition parameters for these experiments at the MRI-linac are provided in Supplementary Table 1.

Spatial resolution was prospectively tested using volunteer imaging both quantitatively and qualitatively for two anatomical sites: the brain and thorax. Additionally phantom imaging was used to investigate hallucinations in the model (Supplementary discussion 1, Supplementary Fig. 3, and Supplementary Table 2).

**Fig. 3:**
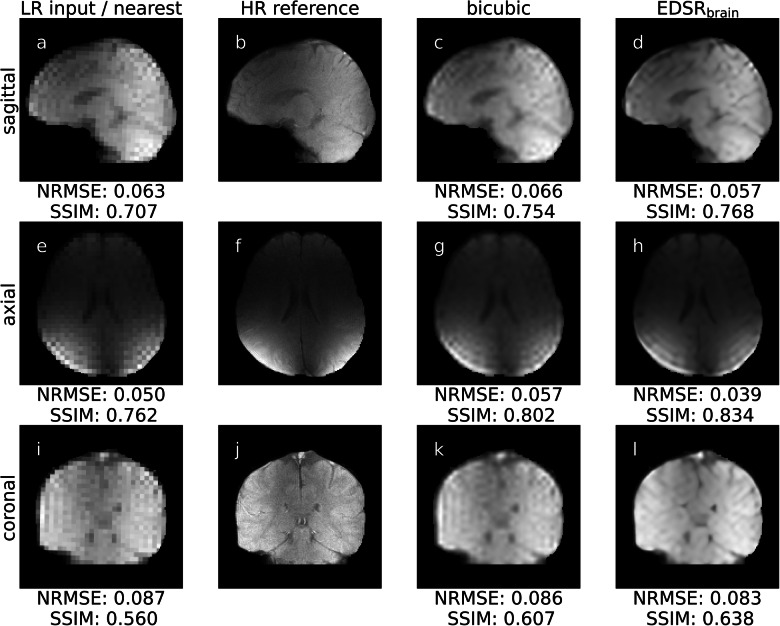
Super-resolution on orthogonal single-image brain MRIs. These MRIs were acquired on a prototype 1.0 T MRI-linac. Quick-to-acquire low spatial resolution acquisitions (LR input / nearest [**a**], [**e**], [**i**]) were input to super-resolution technique bicubic interpolation [**c**], [**g**], [**k**] and EDSR_brain_ [**d**], [**h**], [**l**]. These super-resolution images were then compared to a long-to-acquire high spatial resolution (HR reference [**b**], [**f**], [**j**]) through normalised root mean-square-error (NRMSE) and structural similarity (SSIM). Additionally, the low spatial resolution acquisitions had nearest neighbour applied to allow for quantitative analysis with the high spatial resolution reference. All images scaled between 0 and 1. LR low resolution, EDSR enhanced deep super-resolution, HR high resolution.

**Fig. 4:**
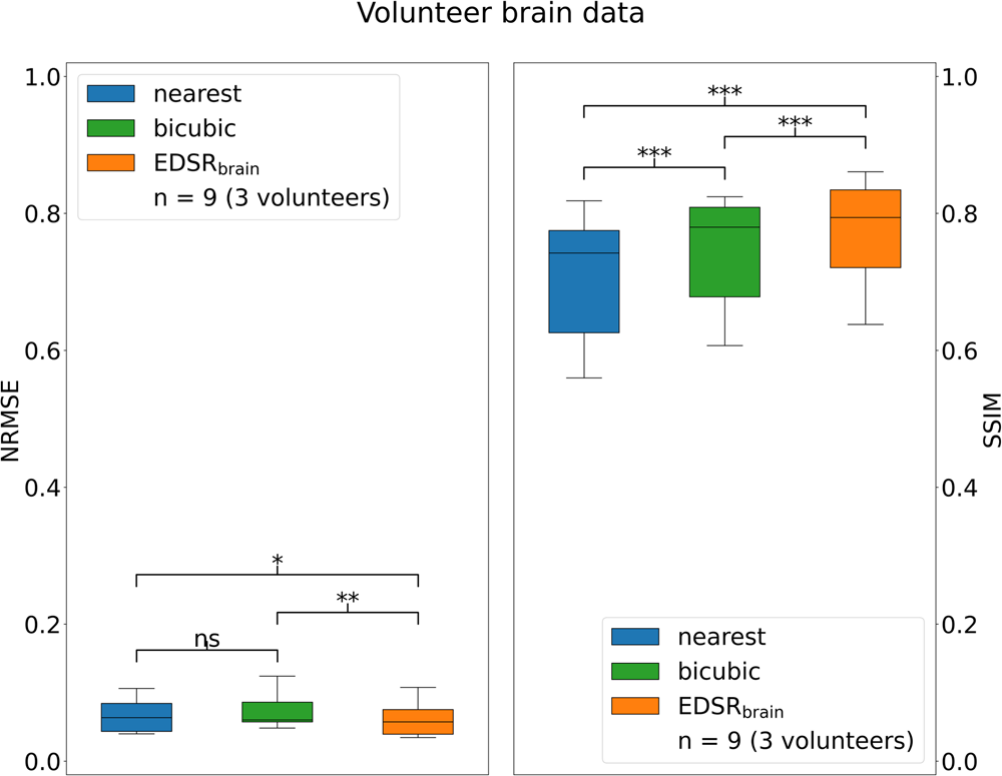
Quantitative performance measures of deployed super-resolution. These results are for volunteer brain imaging on a prototype 1.0 T MRI-linac. Box plots illustrate the distribution of the normalised root mean-square-error (NRMSE) and structural similarity (SSIM) for up-sampling methods. Asterisks denote statistical significance in a paired t test. ns: no statistical significance, *p < 0.05, **p < 0.01, ***p < 0.001. Exact *p* values are given in Supplementary data 1. Error bars correspond to the 1.5× interquartile range values. EDSR enhanced deep super-resolution.

Volunteer brain imaging was performed to test super-resolution on anatomy with limited motion. Low-resolution acquisitions (LR_brain-n_^FLASH-FS-SAGITTAL^, LR_brain-n_^FLASH-FS-AXIAL^, LR_brain-n_^FLASH-FS-CORONAL^ of Supplementary Table 1) were up-sampled utilising the deep learning-based EDSR_brain_ model and bicubic interpolation. A qualitative comparison was made between these up-sampled super-resolution images and their corresponding high-resolution images (HR_brain-n_^FLASH-FS-SAGITTAL^, HR_brain-n_^FLASH-FS-AXIAL^, HR_brain-n_^FLASH-FS-CORONAL^ of Supplementary Table 1). A quantitative comparison between the up-sampled super-resolution images were then compared to their HR counterpart through a NRMSE and SSIM. To remove extreme bright spots in the images, any voxel that was greater than the 99.9th percentile of the entire image was set as the maximum (thus only reassigning 0.1% of voxels). To equalise the noise floor all images were min-max normalised between 0 and their maximum. A misalignment of the low-resolution and high-resolution brain MR images was observed and hypothesised to be due to head motion between serial low-resolution and high-resolution acquisition. Each super-resolution image (nearest neighbour, bicubic interpolation, EDSR_brain_) was rigidly registered to the corresponding high spatial resolution reference image using SimpleITK^44^. Finally, a mask was generated on the reference HR image to isolate the pixels containing the brain utilising thresholding and morphological operations using scikit-image^45^. This reference HR mask was applied jointly to the super-resolution images prior to performance metric calculation. The results for this experiment are displayed in Figs. 3, 4, and Supplementary Figs 4, 5, 6.

**Fig. 5:**
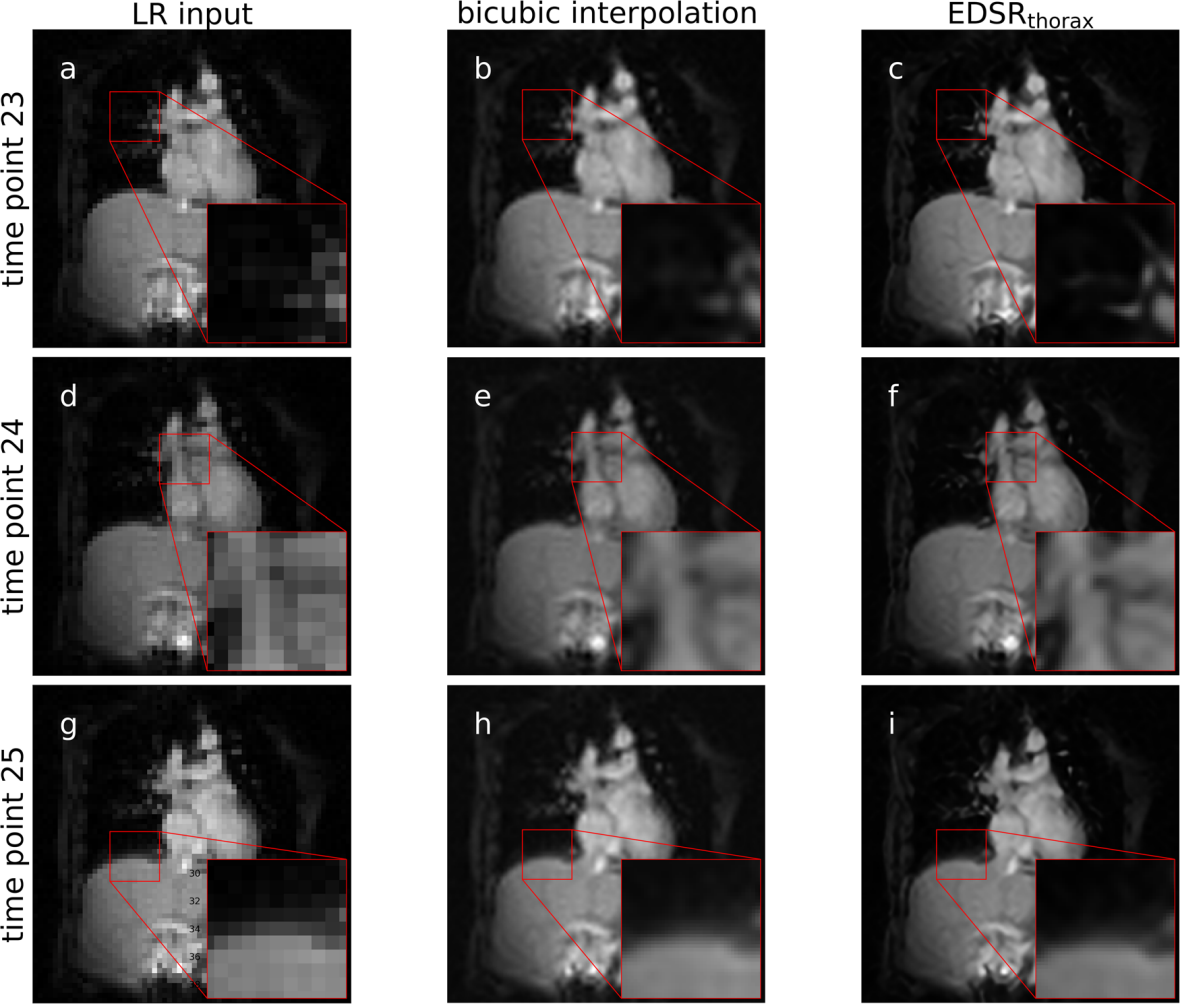
Super-resolution on thorax real-time MRI. These MR images were acquired on a prototype 1.0 T MRI-linac. A joint section of the real-time MRI sequence is displayed. [**a**], [**d**], [**g**] display the quick-to-acquire low spatial resolution input (LR input). These acquisitions were provided to the super-resolution techniques bicubic interpolation [**b**], [**e**], [**h**] and EDSR_thorax_ [**c**], [**f**], [**i**]. Zoomed insets are provided to various anatomical regions to illustrate the image enhancement offered using super-resolution. LR low-resolution, EDSR enhanced deep super-resolution.

Volunteer thorax imaging was undertaken to observe super-resolution on anatomy where high amplitude (due to inhalation/exhalation) and high frequency (due to the cardiac cycle) motion is present. Real-time acquisitions were taken in the coronal imaging plane to clearly observe diaphragm and cardiac motion. A variety of sequences were selected including fast low angle shot (FLASH) and steady state free precession (SSFP) sequences. Due to the inevitable increased acquisition time of high-resolution culminating in motion artifacts and mismatches between the respiratory and cardiac cycle phases, a direct quantitative comparison between super-resolution images and high-resolution images was not undertaken. Reconstructed images with/without super-resolution are displayed in Fig. 5 (LR_thorax-1_^FLASH-FS^ of Supplementary Table 1).

Temporal resolution was evaluated using a motion phantom in a latency experiment. Three performance indicators were measured: end-to-end system latency, geometric error, and latency-corrected geometric error. The end-to-end system latency is the time differential between motion of a tracked target and the MLC adapting the treatment beam to this motion. This is divided into three main categories: MR imaging, target localisation, and MLC tracking^5^. Geometric error is defined by the distance between the centroid of a target (ground-truth) and the centroid of the MLC aperture (prediction). Latency-corrected geometric error is the geometric error with the latency being retrospectively removed thereby separating the error into latency-contributed and tracking-contributed error with the latter being influenced largely by the provided MR images.

The experimental set-up was as follows: an MRI-compatible Quasar one-dimensional motion phantom (Modus Medical Devices, Canada) was placed at isocentre at a source-to-surface distance of 2.4 m. A single angle conformal treatment beam was applied, irradiating a moving spherical target (radius ≈ 30 mm) contained in the motion phantom. To quantify latency and error, electronic portal imaging detector (EPID) images were acquired at 3.6 Hz, directly imaging the treatment beam. The motion phantom was programmed to a sinusoidal trace to quantify latency and subsequently to a patient breathing trace to quantify error. The motion phantom was imaged with an eight-coil receiver array to allow for increased image quality and higher temporal resolution. Acquisition data streamed from the Siemens Image Calculation Environment (ICE) through to Gadgetron (with our integrated framework). MR images were generated in Gadgetron using a GRAPPA-based reconstruction^7^. These reconstructed images were then sent to the tracking software with or without up-sampling within Gadgetron. We tested this on low spatial resolution MRI to establish a baseline latency and error. This was followed by utilising EDSR_thorax_, bicubic interpolation, and high spatial resolution MR images to quantify changes in latency and error compared to the low spatial resolution baseline. The sinusoid and patient trace experiments for each type of imaging (low spatial resolution, EDSR_thorax_, bicubic interpolation, and high spatial resolution) were performed three times consecutively to increase the reliability of the results (Table 1). EPID images were saved for retrospective latency and error analysis.

**Table 1:** Key performance indicators for multi-leaf collimator tracking performance using super-resolution

Up-sample Method	Tracking Resolution	Latency [s]	RMSE [mm]	RMSE_latency-corr_ [mm]
No Up-Sampling	64 × 64	0.350 ± 0.005	3.36 ± 0.06	1.46 ± 0.18
EDSR_thorax_	256 × 256	0.362 ± 0.002	3.33 ± 0.02	0.82 ± 0.04
Bicubic Interpolation	256 × 256	0.348 ± 0.001	3.19 ± 0.06	0.82 ± 0.15
No Up-Sampling	256 × 256	1.626 ± 0.020	N/A	N/A

Results provided are the mean ± standard deviation over three consecutive measurements. EDSR_thorax_ and bicubic interpolation increased the tracking resolution with negligible effect on the latency. The tracking error (RMSE) when the motion phantom target moved to a patient lung tumour trace, decreased using super-resolution. EDSR: enhanced deep super-resolution. RMSE: Root mean-square-error. RMSE_latency-corr_: Root mean-square-error after latency has been removed in post-processing.

**Fig. 6:**
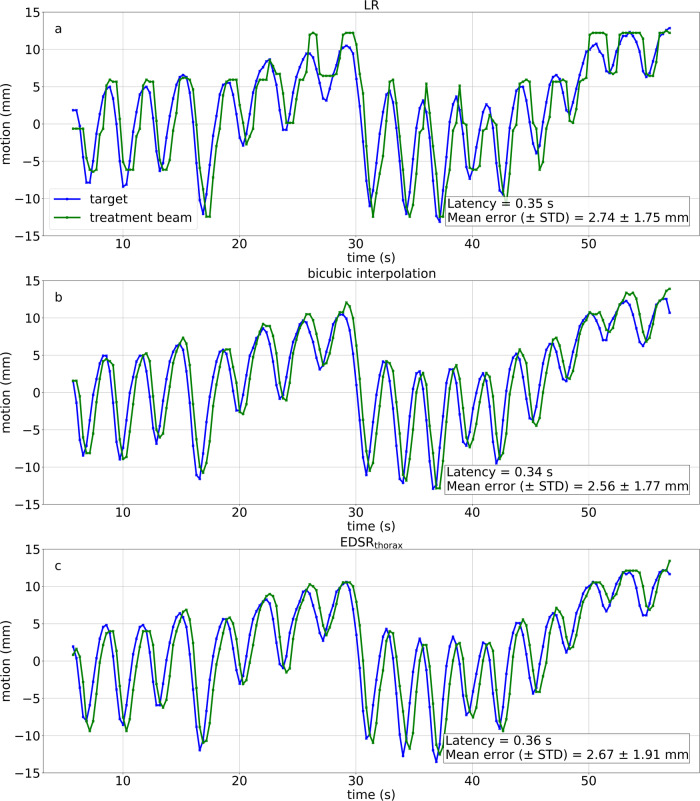
Motion traces of the motion phantom experiment. A target moving to a previously measured patient lung trace was simultaneously measured with the multi-leaf collimator aperture. **a** Motion trace for low resolution imaging. **b** Motion trace for when bicubic interpolation was used. **c** Motion trace for when EDSR_thorax_ was used. Low-resolution (LR) imaging decreased the quality of tracking compared to super-resolution imaging (EDSR_thorax_ and bicubic interpolation). Latency and mean error results are provided for each trace. EDSR enhanced deep super-resolution, STD standard deviation.

The EPID images were processed in a manner consistent with a multi-target tracking experiment on the same MRI-linac^5^. The same pre-defined templates for the aperture and target were used for each type of imaging, ensuring the most valid comparison. The time-series EPID images were run where in each frame a template match was performed for the target and aperture. These templates were then used to calculate a centroid forming a trace. End-to-end system latency was calculated by fitting two sinusoids: one to the target trace and the other to the aperture trace when the motion phantom was moving in a sinusoid. These two sinusoids had a phase shift equal to the end-to-end system latency. Geometric error was calculated by measuring the root mean-square-error between target trace and the aperture trace when using the patient trace. Latency-corrected geometric error was calculated by first shifting the aperture trace by a temporal factor equal to the measured end-to-end system latency then calculating the root mean-square-error between the target trace and this shifted aperture trace. The results from this experiment are presented in Fig. 6 and Table 1.

Volunteer diaphragm imaging was conducted to observe tracking efficacy on human anatomy. For ethical and safety considerations, no radiation was used in this experiment. As a result, a direct latency and subsequently error calculation was not made (cf. latency experiment). We do, however, use the corresponding latency results from the latency experiment due to the acquisitions being extremely similar (supplementary Table 1). Here, we simulated thorax treatment using real-time super-resolution enhanced MLC tracking. This workflow was conducted prospectively at the MRI-linac for testing the integration of super-resolution into the MRI-linac and re-created retrospectively for analysis. This analysis served three purposes: an extra dataset for super-resolution inference using previously untested acquisition parameters, visualising super-resolution enhanced tracking on anatomy, and simulating the effect of latency on tracking anatomy. The results from this experiment are displayed in Supplementary Note 1 and Supplementary Fig. 7.

### Ethics

Prospective volunteer imaging studies on the MRI-linac were performed under the ‘Magnetic Resonance Imaging in healthy volunteers’ study approved by the South Western Sydney Local Health District Human Research Ethics Committee (SWSLHD HREC Reference No: HE 15/270). All volunteers gave written informed consent and were prospectively recruited for validation of the super-resolution methods in this study. The figures these data relate to are Figs. 3, 4, 5, Supplementary Figs 4, 5, 6, 7, in addition to Supplementary data 1 and 2.

Fine-tuning the EDSR_brain_ model to create the EDSR_thorax_ model utilised data from the AVATAR study. The AVATAR study was sponsored by the University of Sydney and was approved by the Hunter New England Local Health District Human Research Ethics Committee (NSW HREC Reference No: HREC/12/HNE/414). The protocol and Participant Information and Consent Form (PICF) both allowed for de-identified data to be shared with the University of Sydney for future research. Therefore, no additional approvals were required to use this data.

Three de-identified datasets from the Cancer Imaging Archive were used to train and evaluate the super-resolution models: QIN-GBM Treatment Response, UPENN-GBM, and Prostate-diagnosis. These datasets are available for approved research use on the Cancer Imaging Archive upon agreement with their data usage policy and restrictions. No additional approvals were necessary. The figures these data relate to are Fig. 2 and supplementary fig. 2 in addition to supplementary data 1.

### Reporting summary

Further information on research design is available in the Nature Portfolio Reporting Summary linked to this article.

## Results

### Super-resolution training and retrospective validation

We begin our study by training enhanced deep super-resolution (EDSR) neural networks to MRI datasets of the human brain (EDSR_brain_) and thorax (EDSR_thorax_) across different sequence types (see methods for full details). EDSR_brain_ (and EDSR_thorax_) had approximately 43 million trainable parameters indicating a large learning capacity. To demonstrate the improved performance of our super-resolution network compared to conventional methods, such as bicubic and nearest-neighbour interpolation, in Fig. 2 we retrospectively applied super-resolution to brain images from an external test dataset (the UPENN-GBM dataset)^33,42^. Performance metrics were significantly better for EDSR_brain_ and across T1w, T2w, and FLAIR sequences compared to bicubic (paired *t* test, p«0.0001, n = 630) and nearest neighbour interpolation (paired *t* test, p«0.0001, *n* = 630). Exact p-values provided in supplementary data 1. On average the EDSR_brain_ network reduced the normalized (min-max) root mean-square-error (NRMSE) by 45% when compared to bicubic interpolation. Qualitatively, fine structures could be recovered using super-resolution with EDSR_brain_ recovering sharper edges to finer structures. The individual data points to produce the boxplots in Fig. 2 are provided in supplementary data 1.

Additionally, a super-resolution network trained on thorax images was applied to a prostate imaging dataset, to test whether trained networks were robust to use on anatomical sites that differed from their training domains (supplementary discussion 1 and Supplementary Fig. 2)^33,43^. Minimal hallucination-type artifacts were observed with this out of domain use and performance metrics were significantly better for EDSR_thorax_ compared to bicubic interpolation (paired *t* test, p«0.0001, *n* = 89) and nearest neighbour (paired *t* test, p«0.0001, *n* = 89). Exact *p* values provided in Supplementary Data 1.

### Deployed super-resolution brain imaging

Having demonstrated the strength of the EDSR method in silico, we now turn to analyze the performance of the trained network in the real-world where AI algorithms can struggle to show performance benefits. We present the results of a suite of healthy volunteer imaging studies that test the performance of super-resolution technologies deployed for prospective use on our 1.0 T MRI-Linac. Bicubic interpolation was selected as a baseline, non-deep learning-based, technique for increasing the spatial resolution of the real-time MRI. Nearest neighbour was also used to quantitatively compare the low spatial resolution images to a high spatial resolution reference. All volunteer imaging studies were performed on the MRI-linac and are therefore external validation datasets as the data used to train the deep learning models were from different imaging centres.

Orthogonal brain imaging on the MRI-linac demonstrated super-resolution in an anatomical area with minimal motion and consequently slow high spatial resolution reference images were obtained (Fig. 3). Three volunteers were imaged forming a dataset of 9 orthogonal slices. We note that while all interpolation techniques have been prospectively deployed on the MRI-Linac, it is only possible to apply one interpolation method in real-time and thus images presented in Fig. 3 were retrospectively processed for comparison of algorithms on the same raw data.

Qualitatively, super-resolution successfully recovered high spatial frequency features showing comparable anatomical detail to the high spatial resolution reference imaging while taking significantly less time to image. Additionally, Gibb’s ringing artifacts were reduced and recovery of fine structure was more prominent when utilising EDSR_brain_ compared to bicubic interpolation. Supplementary Figs 4, 5, 6 provides the results for all three volunteers.

Quantitatively, EDSR_brain_ outperformed both nearest neighbour (paired *t* test, *p* = 0.0127/ < 0.0001 [NRMSE/SSIM], *n* = 9) and bicubic interpolation (paired *t* test, *p* = 0.0019/0.0001 [NRMSE/SSIM], *n* = 9) to statistical significance when measuring the NRMSE and structural similarity (SSIM) as demonstrated in Fig. 4. Exact *p* values provided in Supplementary Data 1. On average the EDSR_brain_ network reduced the NRMSE by 17% for the 1.0 T MRI-Linac experiment when compared to bicubic interpolation, which is smaller than that observed for the external dataset tested in Fig. 2. The individual data points to produce the boxplots in Fig. 4 are provided in supplementary data 1.

### Super-resolution thorax imaging

Respiratory motion can be responsible for the motion of radiotherapy targets across the cardiothoracic and abdominal regions including the lungs, liver, and pancreas^46^. Pursuant to this, we performed a series of breath-hold and free-breathing thorax and abdomen real-time MRI studies to observe the effect of super-resolution in these anatomical regions. Our pipeline, upon image reconstruction in Gadgetron, immediately applied super-resolution to the real-time MR images while only imposing an additional 5 ms processing time on an NVIDIA RTX A5000 GPU workstation (NVIDIA, USA). Using an up-sample factor of 4, pixel sizes were reduced from 6.5 × 6.5 mm^2^ to 1.56 × 1.56 mm^2^ leading to an increase in anatomical detail in the cardiothoracic region (Fig. 5).

Qualitatively, integrated super-resolution produced real-time MR images with sharper anatomical boundaries with this effect most noticeable using EDSR_thorax_ as opposed to bicubic interpolation (Fig. 5). Due to the prohibitive increase in the acquisition time, a high spatial resolution reference for a quantitative comparison was not attainable for cardiothoracic real-time MRI. Supplementary data 2 contains animated GIFs of all thorax cine acquisitions.

### Real-time adaptive MRI-guided radiation therapy integration

The time to perform inference with the EDSR networks was measured to be approximately 5 ms using the internal system clock, which is suitable for real-time applications and similar to the variability in end-to-end latency of the system (Table 1). Real-time adaptive MRIgRT was experimentally tested on the MRI-linac using our integrated framework, a motion phantom, and MLC tracking. Two performance indicators were measured: the latency and tracking error. As measured by an electronic portal imaging detector (EPID), the latency of the integrated system remained consistent with other published baselines when utilising super-resolution for 4 Hz real-time MRI^5,26,47^. Super-resolution marginally decreased tracking error with bicubic interpolation showing the least error, however, after accounting for latency in post-processing both EDSR_thorax_ and bicubic interpolation showed approximately 40% less error. Quantitative results for these experiments are detailed in Table 1. Upon moving the motion phantom’s target to a previously measured patient lung tumour trace, super-resolution assisted in the accurate and precise placement of the MLC aperture over the target (Fig. 6).

Diaphragm tracking of a volunteer was performed to observe the effect of super-resolution on tracking a thoracic site. Retrospectively, real-time MRIs of the thorax were tracked using a template matching approach for consistency with the deployed tracking on the MRI-linac. We observed increased granularity in the predicted diaphragm positions owing to the reduced pixel size afforded by super-resolution. Adding a latency offset (corresponding to experimentally measured latencies) to the tracked diaphragm positions demonstrated the suitability of using super-resolution techniques for tracking anatomy (supplementary note 1 and supplementary fig. 7). High resolution real-time MRI was inappropriate for real-time guidance applications due to the considerable latency increase leading to inaccurate template positioning of the diaphragm.

## Discussion

Deep learning-based super-resolution was integrated into the real-time adaptive MRIgRT workflow. Our experiments demonstrate a significant increase in the spatial resolution without reducing the temporal resolution thus effectively increasing the spatiotemporal resolution.

We demonstrated this prospectively in two anatomical sites: the brain and thorax. It was previously reported in a multi-target tracking experiment on the same MRI-linac, the pixel size of the real-time MRI used for tracking was 3.125 × 3.125 mm^2^ for 4 Hz imaging^5^. In an evaluation of intrafraction and interfraction tumour motion seen in lung stereotactic body radiation therapy on a MRIdian ViewRay MRI-linac (ViewRay Inc., USA), real-time MRI was used with a pixel size of 3.5 × 3.5 mm^2^ for 4 Hz imaging^48^. In our work, after up-sampling, the pixel size was 1.56 × 1.56 mm^2^. Anatomical features/edges were clearer/sharper when using super-resolution compared to using low spatial resolution images. Comparing deep learning-based super-resolution with bicubic interpolation indicated a performance boost when using the former method. This was demonstrated through improved full-reference image quality metrics (Figs. 2, 4) and the qualitative improvement in the recovery of high spatial frequency features seen in Figs. 2, 3, 5.

We note difficulty in acquiring paired low and high spatial resolution anatomical images due to motion between (or during) acquisitions. In the case of brain imaging where single-image orthogonal views were taken, a head displacement between serial low and high spatial resolution MR images could result in a different slice of anatomy being imaged (Fig. 3). The magnitude of motion has been previously investigated in head MRI and is approximately 3 mm per minute^49^. Further investigation of the spatial resolution on anatomy could include immobilisation devices to reduce this motion. The lack of a reference high spatial resolution real-time MRI of the thorax is unavoidable due to phase differences in the respiratory and cardiac cycle leading to changes in diaphragm position and cardiac shape.

While testing our models on external datasets provides useful insights to the robustness of our models, they may provide artificially optimistic performance metrics as the inputs are synthetically created from the labels. We hypothesize this is a reason the results differ between using measured low spatial resolution images (e.g., Fig. 3) and synthetically derived low spatial resolution images (e.g., Fig. 2). This idea has been explored for super-resolution and MR image reconstruction more broadly^50,51^. To demonstrate clinical utility, the repeatability, reproducibility, and robustness of super-resolution methods, especially deep learning-based, should be evaluated across a broad patient cohort on real-world paired data.

For the brain experiments, the enhanced deep super-resolution (EDSR) model was trained on conventional anatomical MRIs from diagnostic 3.0 T MRI scanners. In silico testing of the EDSR model on similar 1.5/3.0 T diagnostic MRI scans (Fig. 2) showed bigger improvements in image quality than the real-world tests of super-resolution on our 1.0 T MRI-linac. We note this performance difference could be explained by the domain shift between the high-field anatomical imaging used for training and the lower-field real-time imaging performed on our MRI-linac. In future, we expect to be able to utilize growing registries of MRI-linac imaging data for training and translation, which would help alleviate this domain shift^52^. However, we also note that in silico tests often overestimate the performance of real-world super-resolution, and that improvements in image degradation processes for network training may improve real-world performance^53^. Furthermore, real-time MR imaging can lead to low signal-to-noise ratio (SNR) images, especially on lower-field systems, and future networks may be adapted to minimise the impact of this noise on super-resolution performance^24,54^.

Hallucinations, in the context of MRI super-resolution, are incorrect (usually high frequency) features introduced by the prior that cannot be produced from the measurements^55^. Incorrect features could be especially problematic in the field of real-time adaptation where a human observer may not be able to detect and correct for these as radiation is being delivered to the patient. We provide a hallucination analysis in supplementary discussion 1. The summary of this analysis is that the EDSR-based methods held good quantitative performance metrics on data well outside the training domain (prostate and phantom MRIs c.f., brain and thorax). Distortions of a multi-compartment phantom were apparent in all up-sampling methods (bicubic and EDSR-based). Noting that hallucinations can be more pronounced in super-resolution applications when the learned probability distribution (brain/thorax) does not match the images on which the network is being tested (phantom), the EDSR-based methods uniquely sharpened the distorted phantom edges. While we did not observe any clear hallucinations while testing EDSR networks on brains and thoraxes, we note that there is a domain shift between high-field training and low-field deployment regimes. Future work will likely integrate task-informed hallucination maps into the real-time deployment, further building confidence in super-resolution images before use for guidance in patient treatments can be considered^55^.

While balanced steady state free precession (bSSFP) sequences are preferred for real-time imaging due to high spatiotemporal resolution, we note that banding artifacts will change appearance as sequences parameters such as repetition time (TR) are adjusted to alter the imaging resolution and that such changes should be accounted for in clinical deployment of super-resolution technology^56^.

Our integration of super-resolution did not prohibitively increase the tracking latency. On the same MRI-linac, in a multi-target tracking experiment, the uncertainty in the latency was measured to be 40 ms^5^. Our measured latency results when low spatial resolution or super-resolution imaging was used were within a 40 ms range (Table 1). We conclude the increased spatial resolution came at a negligible cost to the latency. A comparison can be drawn from previously published experimental results utilising clinically deployed MRI-linacs. Volumetric modulated arc therapy (VMAT) was experimentally demonstrated on an Elekta Unity MRI-linac (Elekta AB, Sweden) utilising multi-leaf collimator (MLC) tracking on real-time MRI as its real-time treatment adaptation strategy^26^. Here, they measured the end-to-end latency (using MLC tracking) to be 328 ms. Characterisation of MLC tracking performance on an Elekta Unity MRI-linac (Elekta AB, Sweden) found latencies of 347 ms and 205 ms for 4 and 8 Hz imaging respectively^47^. We measured a latency of approximately 350 ms using our integrated system that is comparable to clinically deployed systems.

Super-resolution methods offered a slight increase in geometric accuracy as defined by the root mean-square-error (RMSE) with bicubic interpolation offering the largest increase. After correcting for latency, both EDSR_thorax_ and bicubic interpolation reduced the RMSE by around 40% (Table 1). Tracking a spherical target within a motion phantom provides preliminary geometric accuracy results, however, several key differences remain when comparing the experimental set-up to tracking anatomy. These key differences include but are not limited to out-of-plane motion, target deformations, and surrounding anatomy. Further investigation is warranted in this respect. We note that our Gadgetron-based integration also supports neural network frameworks that operate on k-space data to correct motion and distortion, which will likely also improve tracking accuracy^57,58^.

We establish super-resolution integrated into real-time MRI treatment guidance. To the best of our knowledge, this is a world-first in the deployment of deep learning to improve real-time adaptive MRIgRT. We make our framework publicly available to streamline future development (see Code Availability).

### Supplementary information

Supplementary Information

Description of Additional Supplementary Files

Supplementary Data 1

Supplementary Data 2

Reporting Summary

## Data Availability

QIN-GBM Treatment Response (https://wiki.cancerimagingarchive.net/display/Public/QIN + GBM+Treatment+Response), UPENN-GBM (https://wiki.cancerimagingarchive.net/pages/viewpage.action?pageId=70225642), and Prostate-Diagnosis (https://wiki.cancerimagingarchive.net/display/Public/PROSTATE-DIAGNOSIS) are available on the Cancer Imaging Archive for approved research use upon agreement with their respective terms of use and license^31,33,42,43^. AVATAR and MRI-linac volunteer data cannot be shared publicly at this time. The terms of patient/volunteer consent for these studies allow for de-identified data to be shared with external research institutions under a formal collaboration agreement. Researchers wishing to access these data should contact the corresponding author. The data points plotted in Figs. 2, 4 and supplementary fig. 2 can be found in supplementary data 1.

## Code availability

Integration code is available at https://github.com/Image-X-Institute/MRI-Linac_SuperResolution. This repository is accessible and archived at Zenodo^59^.
