# Supplementary material for: Super-resolution neural networks improve the spatiotemporal resolution of adaptive MRI-guided radiation therapy
Source: Commun Med (Lond). 2024 Apr 4;4:64. doi: 10.1038/s43856-024-00489-9 (PMC10994938; doi:10.1038/s43856-024-00489-9)
Supplement: Supplementary file 1 — Supplementary Information [file 43856_2024_489_MOESM1_ESM.pdf]

Supplementary method 1 – Deep learning pipeline

We trained two deep learning models corresponding to two anatomical regions, the brain and the thorax. This training pipeline is outlined in supplementary figure 1.

An edge-based loss function was used to recover high frequency features lost by the cropping of the periphery of k-space. This is detailed in equation 1.

Equation 1: Edge-based L1 loss computation.

$$\mathrm{EdgeBasedL1Loss} = \alpha \cdot \mathrm{loss}_{\mathrm{pixels}} + (1 - \alpha) \cdot \mathrm{loss}_{\mathrm{edges}} \qquad (\alpha = 0.01)$$

$$\mathrm{loss}_{\mathrm{pixels}} = \overline{\left|\mathrm{y}_{\mathrm{label}} - \mathrm{y}_{\mathrm{prediction}}\right|_{l1}}, \qquad \mathrm{loss}_{\mathrm{edges}} = \overline{\mathrm{M}\left|\mathrm{y}_{\mathrm{label}} - \mathrm{y}_{\mathrm{prediction}}\right|_{l1}}$$

$$\mathrm{M} = \mathrm{Canny}(\mathrm{y}_{\mathrm{label}}, \mathrm{threshold}_1, \mathrm{threshold}_2)$$

$$\mathrm{threshold}_1 = \mathrm{percentile}(\mathrm{y}_{\mathrm{label}}, 90), \qquad \mathrm{threshold}_2 = 0.4 \cdot \mathrm{threshold}_1$$

Consider two n×n matrices representing a label high-resolution (y_{label}) and a predicted super-resolution image ($y_{prediction}$). The edge-based l1 loss (EdgeBasedL1Loss) is the linear combination of the l1 loss of the total matrices ($loss_{pixels}$) and the l1 loss of the masked matrices ($loss_{edges}$). The weighting term (α) was assigned 0.01 empirically from the QIN-GBM treatment response validation data. The n×n mask M, is generated using a Canny filter of y_{label}, extracting the edges of the label high-resolution image. Subsequently, when applied as a mask, isolates the loss calculation to these edges. This Canny filter utilises a high ($threshold_1$) and a low ($threshold_2$) value for hysteresis thresholding. These high and low threshold values were selected empirically based off the 90th percentile of y_{label}. During training and validation, this calculation is performed over the batch, where y_{label}, $y_{prediction}$, and M are b×n×n matrices (b, batch size) where M contains a unique edge map for each pair of images in the batch. Adapted from previously published work on single image super-resolution[36].

Supplementary discussion 1 – Hallucination analysis.

Hallucinations are a problem in deep learning for medical imaging reconstruction and enhancement. It is important models are not generating features that are not there. We test for hallucination phenomena by applying our super-resolution networks to images that are significantly outside the training domain of brain and thoracic images (a grid phantom and an external database of prostate MRIs).

A NIST system standard model 130 phantom (National Institute of Standards and Technology, USA) was volumetrically imaged at both a low spatial resolution ($LR_{phantom}^{FLASH\text{-}FS}$, see supplementary table 1 for acquisition parameters) and high spatial resolution ($HR_{phantom}^{FLASH\text{-}FS}$) at the 1.0 T MRI-linac. In this instance, negligible motion between low-resolution and high-resolution acquisitions was present. The centre 25 low-resolution slices were input to super-resolution technique and compared to their corresponding high-resolution slices through a normalised (min-max) root mean-square-error (NRMSE), and structural similarity (SSIM).

This analysis was performed on the MRI-linac's vendor reconstructed DICOM images to isolate the performance of the super-resolution techniques themselves with no post-processing (cf. correction for brain misalignment in the brain experiment). The centre 25 slices were circularly masked to constrain the performance metric calculation to pixels within the phantom and to not contaminate performance calculation with differences in the noise floor. The results are presented in supplementary figure 3.

Both $EDSR_{brain}$ and $EDSR_{thorax}$ showed little hallucinations in this experiment (supplementary figure 3) and produced images that had numerically similar metrics indicating stability in model fine-tuning. This indicates a level of robustness in the developed models. Statistical results are provided in supplementary table 2.

We do observe distortion artifacts in the NIST phantom images for all up-sampling methods (e.g., circles appearing as diamonds in [g], [h], [i] in supplementary figure 3). The visual appearance of these artifacts differs between bicubic interpolation and EDSR methods due to the sharpening of edges performed by the EDSR network.

We additionally ran inference on $EDSR_{thorax}$ on the prostate-diagnosis dataset from the Cancer Imaging Archive[33,43]. The testing pipeline is described in the main methods section. The results for this experiment are provided in supplementary figure 2.

$EDSR_{thorax}$ displayed promising results (outperforming nearest neighbour and bicubic interpolation) in this experiment while showing minimal hallucinations even on an external validation dataset. The

individual data points to produce the boxplots in supplementary figure 2 are provided in supplementary data 1.

For volunteer imaging on the MRI-linac we note minimal presence of hallucinations, however, EDSR methods tended to up-sample artifacts in the low spatial resolution input. This was especially noticeable for the steady state free precession (SSFP) thorax sequence in supplementary data 2. Banding artifacts were simply up-sampled, not removed, when using EDSR methods indicating a lack of artifact removal for SSFP sequences. We do note, however, Gibb’s ringing artifacts in the brain sequences were minimised using EDSR methods. Future implementations could include artifact reduction techniques and/or explicit data consistency.

Supplementary note 1 – Diaphragm tracking.

Here, we present the results from our diaphragm tracking experiment. Part of the diaphragm was tracked using template matching and offset temporally using the measured latency from the beam tracking experiment (table 1). MRI acquisition parameters for supplementary figure 7 are in supplementary table 1 under experiment Thorax-3.

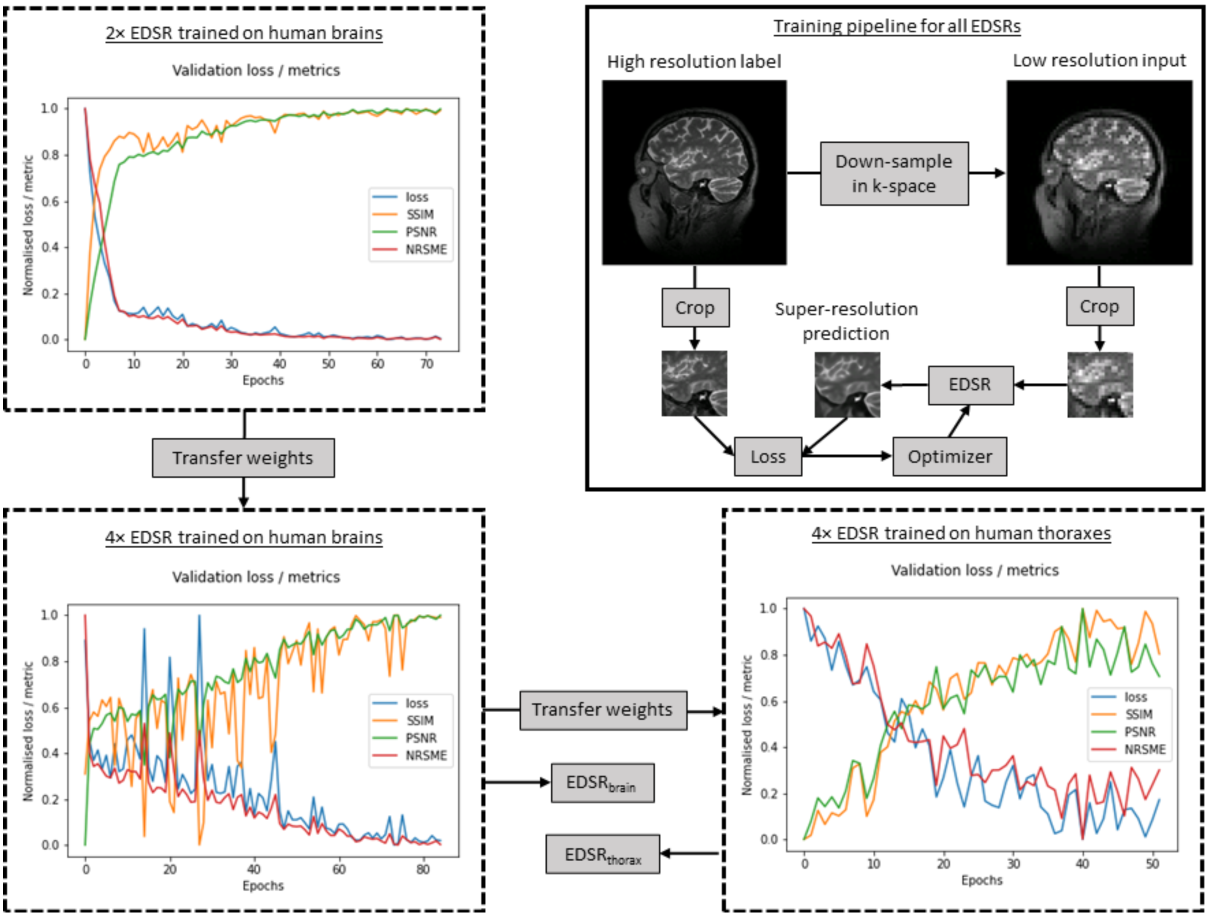

Supplementary figure 1: The deep learning-based super-resolution development. High spatial resolution labels were down-sampled in k-space producing low spatial resolution inputs. Joint random crops were applied to the label input pairs. Cropped inputs were provided to the enhanced deep super-resolution (EDSR) neural network that subsequently made a super-resolution prediction. These predictions were compared through an edge-based loss function and provided to an Adam optimiser utilising a one-cycle learning rate scheduler to update the model parameters. An initial 2× brain model was trained that was then transferred to a 4× brain model for further training. This formed a brain-specific super-resolution model (EDSR$_{brain}$) that was finally fine-tuned to MR images of the human thorax creating a thorax-specific super-resolution model (EDSR$_{thorax}$). SSIM: structural similarity. PSNR: peak signal-to-noise ratio. NRMSE: normalised root mean-square-error.

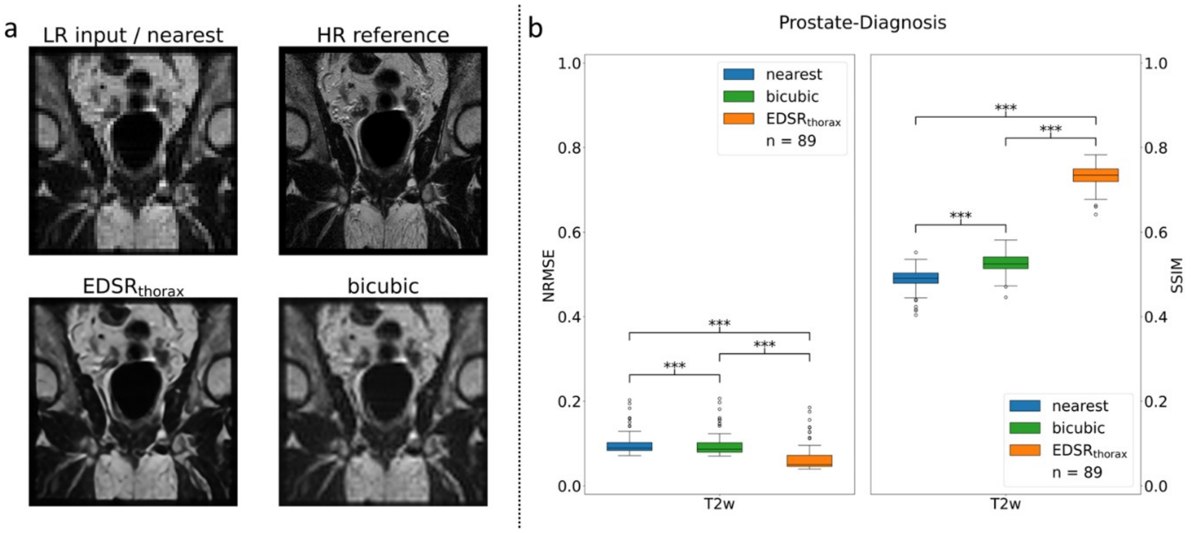

Supplementary figure 2: External validation on prostate-diagnosis dataset. (a) is an example T2w image from prostate-diagnosis. LR input / nearest is the low spatial resolution input to super-resolution technique ($EDSR_{thorax}$ and bicubic) derived from the high spatial resolution label (HR reference). (b) is a boxplot of quantitative performance measures across the n=89 subjects each containing a T2w coronal image. All images scaled between 0 and 1. Asterisks denote statistical significance in a paired t-test. ns: no statistical significance, *: $p < 0.05$, **: $p < 0.01$, ***: $p < 0.001$. Exact p-values are given in supplementary data 1. Error bars correspond to the 1.5× interquartile range values. EDSR: enhanced deep super-resolution, NRMSE: normalised root mean-square-error, SSIM: structural similarity.

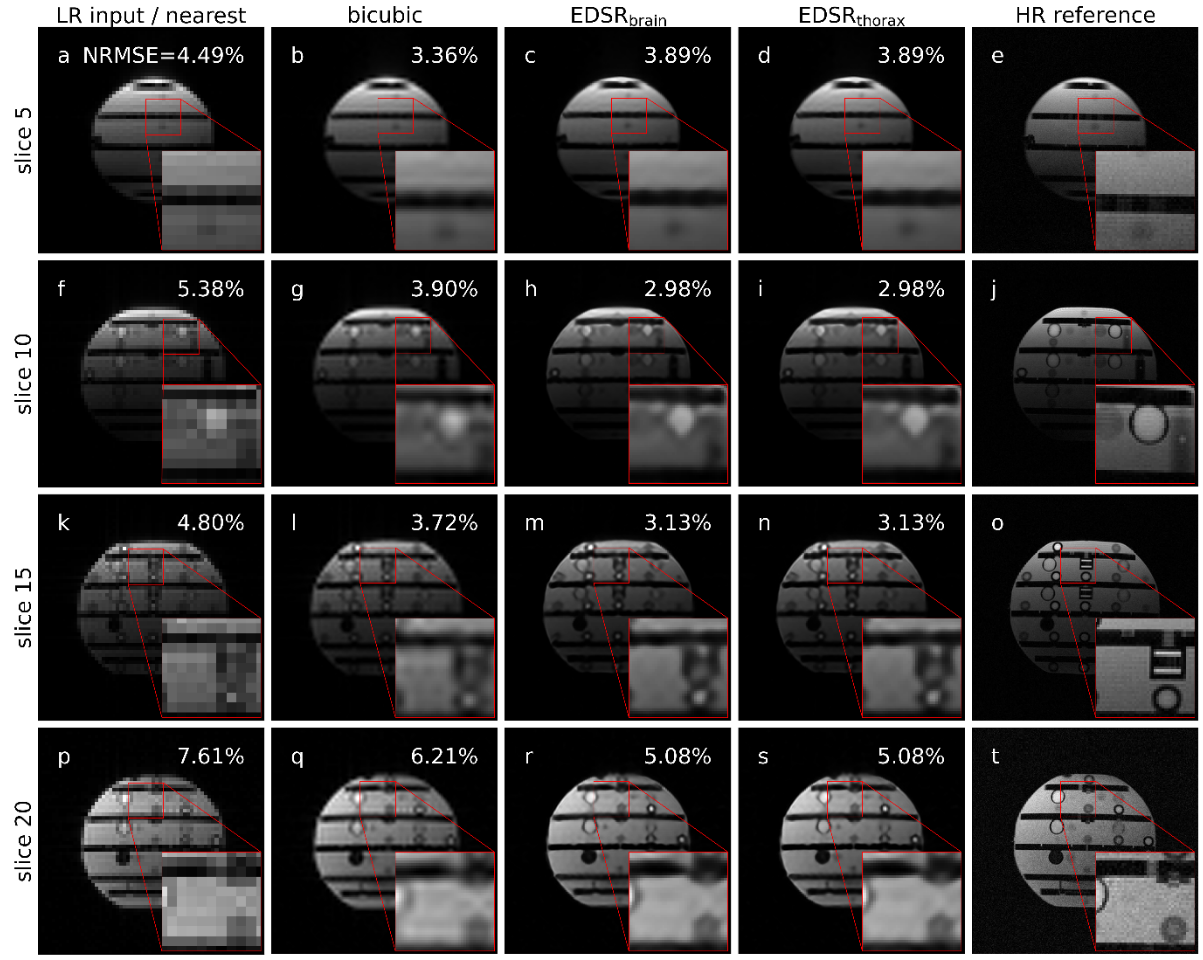

Supplementary figure 3: Super-resolution on a NIST phantom. Low spatial resolution (LR) images were up-sampled using $EDSR_{brain}$, $EDSR_{thorax}$, and bicubic interpolation methods recovering details present in high spatial resolution (HR) reference images. Zoomed insets display the recovery of high spatial frequency features when using $EDSR_{brain}$, $EDSR_{thorax}$, and bicubic interpolation methods. Additionally, the low spatial resolution acquisitions had nearest neighbour interpolation applied to allow for quantitative analysis (NRMSE value) with the high spatial resolution reference. All images scaled between 0 and 1. LR: low-resolution. EDSR: enhanced deep super-resolution. HR: high resolution. NRMSE: normalised root mean-square-error.

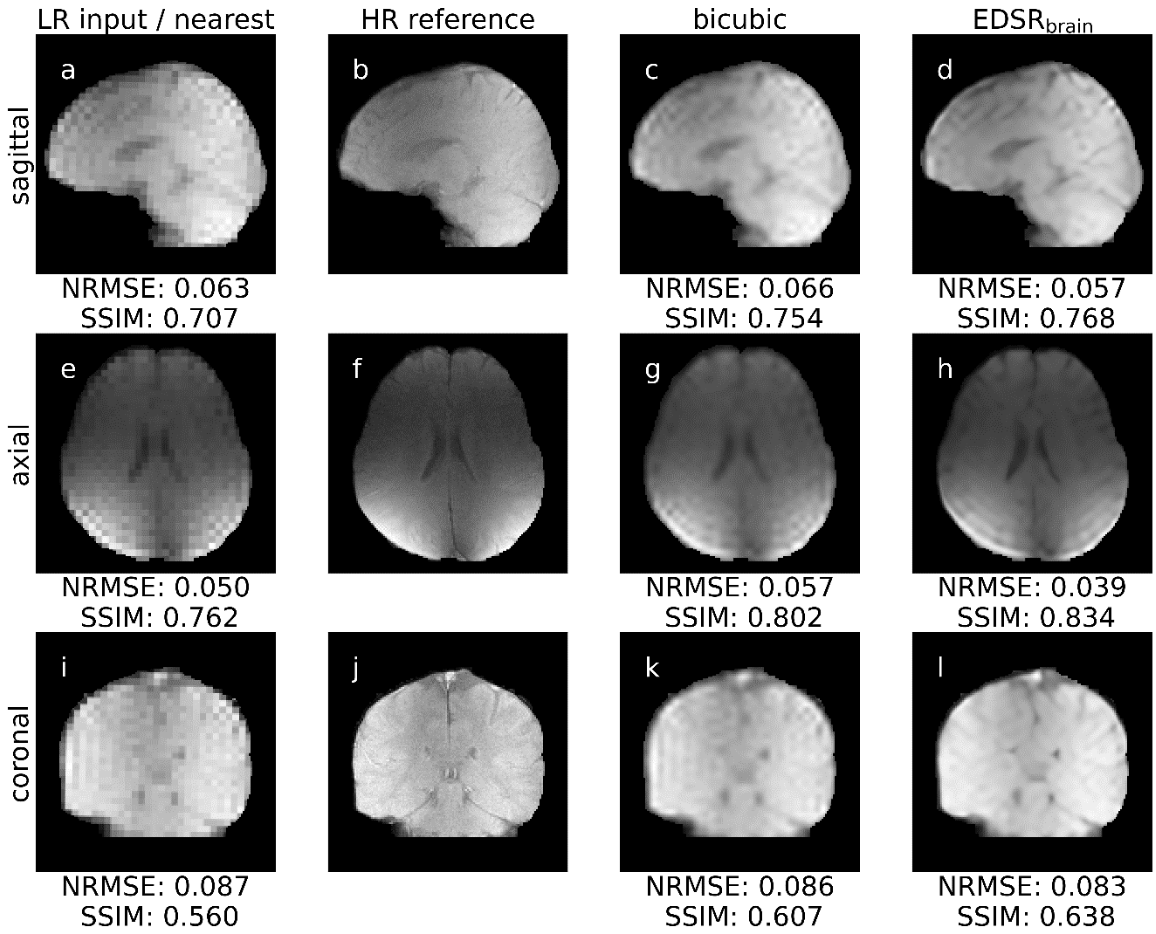

Supplementary figure 4: Super-resolution on orthogonal single-image brain MRIs. *These MRIs were acquired on a prototype 1.0 T MRI-linac. Quick-to-acquire low spatial resolution acquisitions (LR input / nearest [a], [e], [i]) were input to super-resolution technique bicubic interpolation [c], [g], [k] and EDSR$_{brain}$ [d], [h], [l]. These super-resolution images were then compared to a long-to-acquire high spatial resolution (HR reference [b], [f], [j]) through normalised root mean-square-error (NRMSE) and structural similarity (SSIM). Additionally, the low spatial resolution acquisitions had nearest neighbour applied to allow for quantitative analysis with the high spatial resolution reference. All images scaled between 0 and 1. LR: low resolution. EDSR: enhanced deep super-resolution. HR: high resolution.*

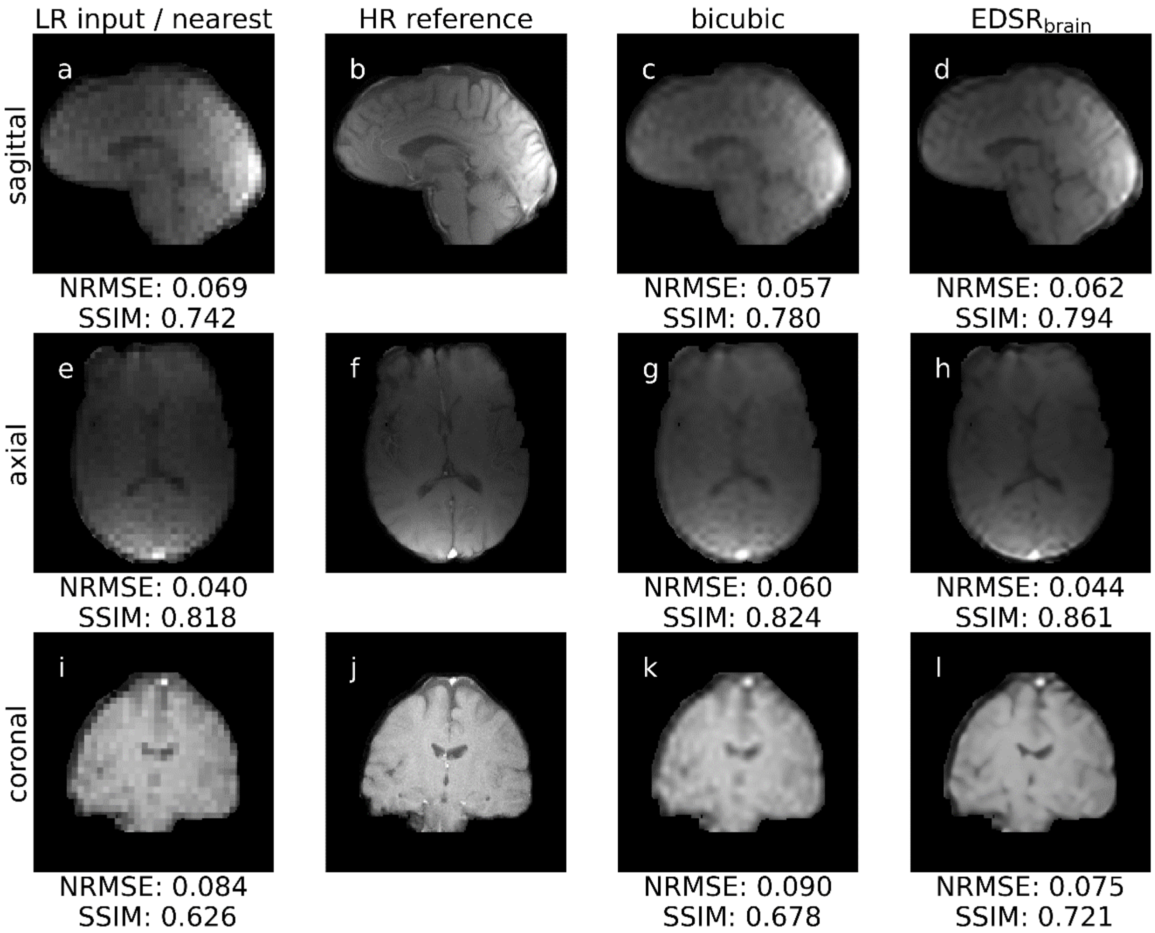

Supplementary figure 5: Super-resolution on orthogonal single-image brain MRIs. These MRIs were acquired on a prototype 1.0 T MRI-linac. Quick-to-acquire low spatial resolution acquisitions (LR input / nearest [a], [e], [i]) were input to super-resolution technique bicubic interpolation [c], [g], [k] and $EDSR_{brain}$ [d], [h], [l]. These super-resolution images were then compared to a long-to-acquire high spatial resolution (HR reference [b], [f], [j]) through normalised root mean-square-error (NRMSE) and structural similarity (SSIM). Additionally, the low spatial resolution acquisitions had nearest neighbour applied to allow for quantitative analysis with the high spatial resolution reference. All images scaled between 0 and 1. LR: low resolution, EDSR: enhanced deep super-resolution, HR: high resolution.

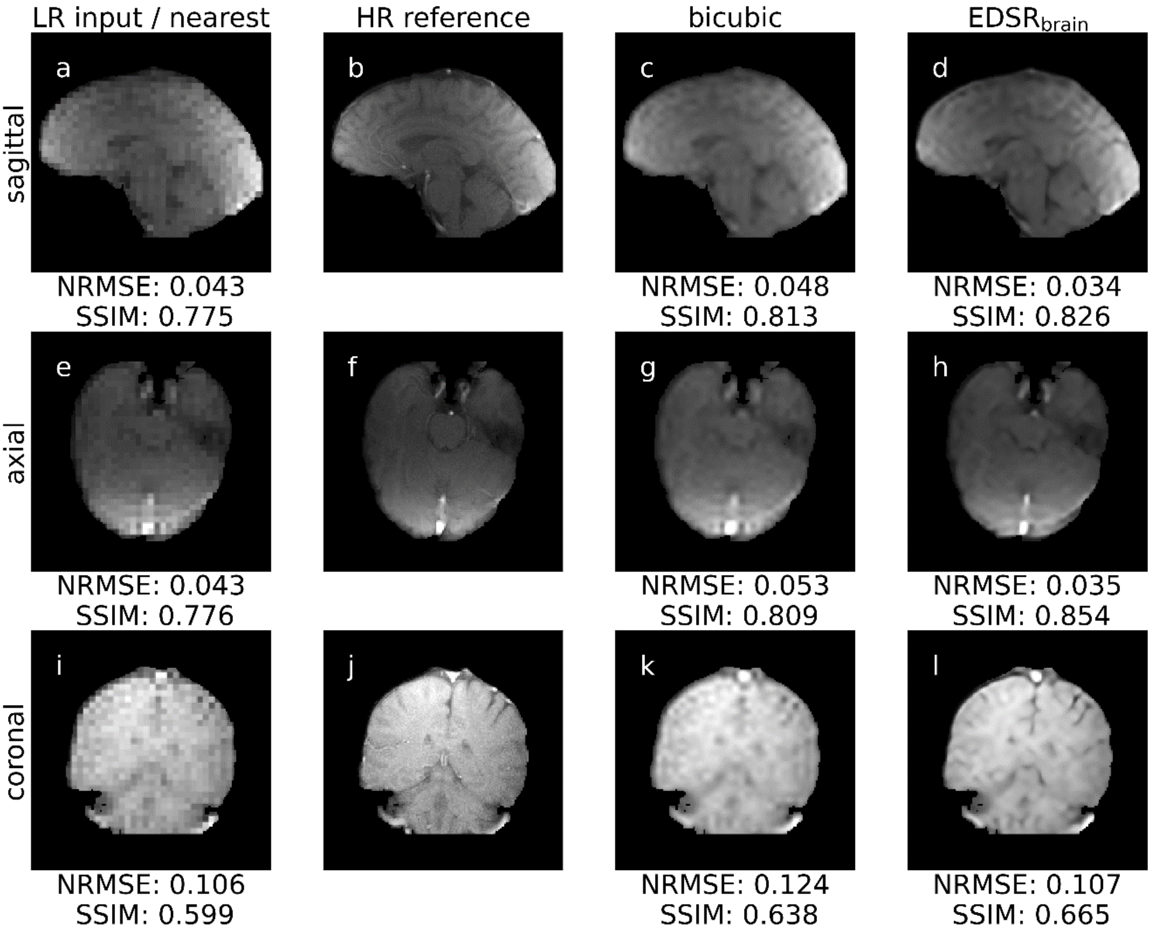

Supplementary figure 6: Super-resolution on orthogonal single-image brain MRIs. These MRIs were acquired on a prototype 1.0 T MRI-linac. Quick-to-acquire low spatial resolution acquisitions (LR input / nearest [a], [e], [i]) were input to super-resolution technique bicubic interpolation [c], [g], [k] and $EDSR_{brain}$ [d], [h], [l]. These super-resolution images were then compared to a long-to-acquire high spatial resolution (HR reference [b], [f], [j]) through normalised root mean-square-error (NRMSE) and structural similarity (SSIM). Additionally, the low spatial resolution acquisitions had nearest neighbour applied to allow for quantitative analysis with the high spatial resolution reference. All images scaled between 0 and 1. LR: low resolution, EDSR: enhanced deep super-resolution, HR: high resolution.

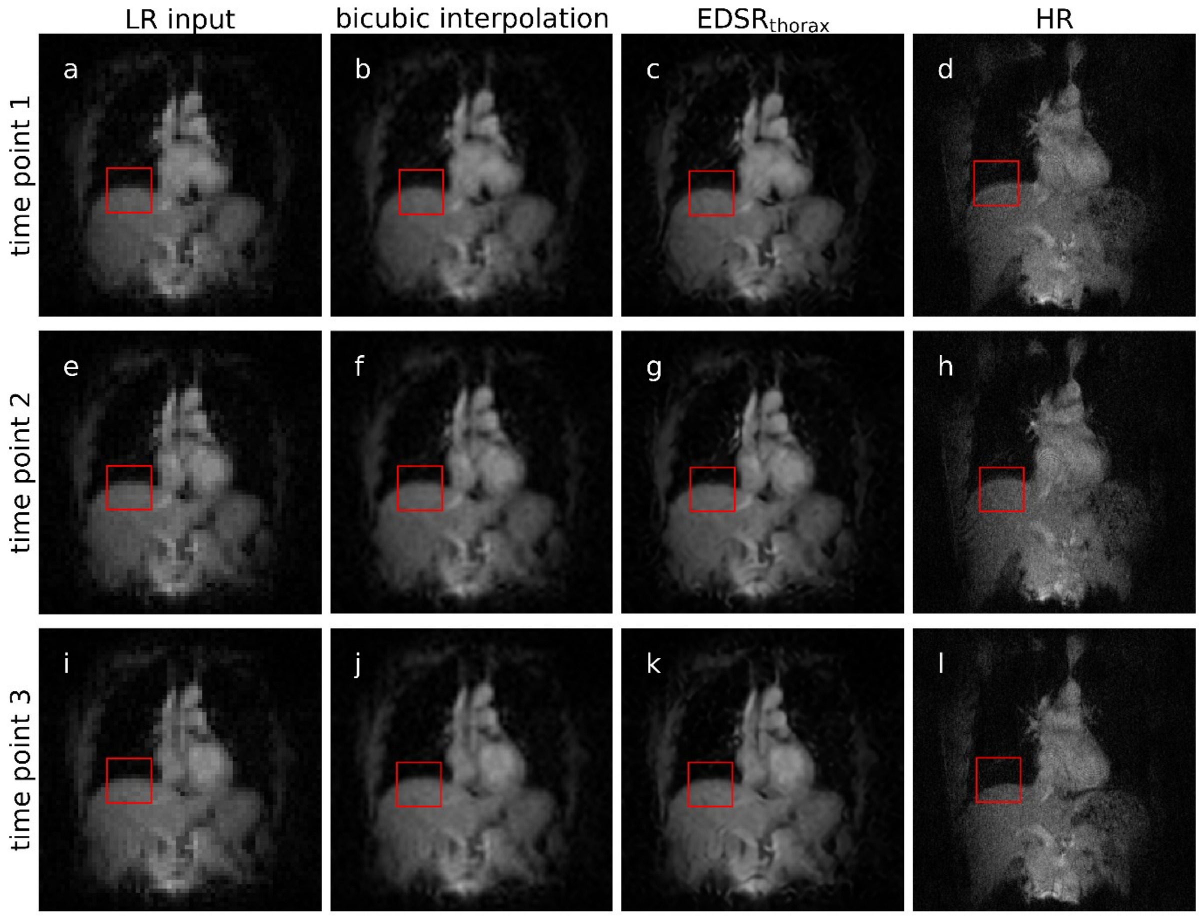

Supplementary Figure 7: Tracking the diaphragm of a volunteer using super-resolution. Red boxes delineate the tracked diaphragm and were offset temporally by the latency previously measured. Tracking increased spatial granularity using $EDSR_{thorax}$ and bicubic interpolation owing to reduced pixel sizes compared to tracking using low-resolution imaging. High spatial resolution imaging produced large latencies manifesting in significant lagging of the template with respect to the diaphragm. LR: low-resolution, EDSR: enhanced deep super-resolution, HR: high resolution.

Supplementary table 1: **Acquisition parameters of experiments.** Acceleration refers to the under-sampling factor, auto-calibration signal width was 16 for acquisitions with acceleration. ^: centre excitation used for quantitative analysis. TR: repetition time. TE: echo time. LR: low-resolution. HR: high-resolution. FLASH: fast low angle shot. SSFP: steady-state free precession.

Experiment	Name	Plane	Matrix size	Field of view (x, y, z) [mm]	TR [ms]	TE [ms]	Sequence type	Number of Excitations	Acceleration
Brain-1	$LR_{brain-1}^{FLASH-FS-SAGITTAL}$	Sagittal	64×64	300, 300, 10	9.34	5.18	FLASH	20^	None
	$HR_{brain-1}^{FLASH-FS-SAGITTAL}$	Sagittal	256×256	300, 300, 10	16.06	9.04	FLASH	20	None
	$LR_{brain-1}^{FLASH-FS-AXIAL}$	Axial	64×64	300, 300, 6.5	10.22	6.06	FLASH	20^	None
	$HR_{brain-1}^{FLASH-FS-AXIAL}$	Axial	256×256	300, 300, 6.5	16.06	9.04	FLASH	20	None
	$LR_{brain-1}^{FLASH-FS-CORONAL}$	Coronal	64×64	300, 300, 6.5	10.22	6.06	FLASH	20^	None
	$HR_{brain-1}^{FLASH-FS-CORONAL}$	Coronal	256×256	300, 300, 6.5	16.06	9.04	FLASH	20	None
Brain-2	$LR_{brain-2}^{FLASH-FS-SAGITTAL}$	Sagittal	64×64	300, 300, 10	9.24	5.13	FLASH	16^	None
	$HR_{brain-2}^{FLASH-FS-SAGITTAL}$	Sagittal	256×256	300, 300, 10	15.97	9.00	FLASH	32	None
	$LR_{brain-2}^{FLASH-FS-AXIAL}$	Axial	64×64	300, 300, 10	9.24	5.13	FLASH	16^	None
	$HR_{brain-2}^{FLASH-FS-AXIAL}$	Axial	256×256	300, 300, 10	15.96	9.00	FLASH	32	None
	$LR_{brain-2}^{FLASH-FS-CORONAL}$	Coronal	64×64	300, 300, 10	9.24	5.13	FLASH	16^	None
	$HR_{brain-2}^{FLASH-FS-CORONAL}$	Coronal	256×256	300, 300, 10	15.96	9.00	FLASH	32	None
Brain-3	$LR_{brain-3}^{FLASH-FS-SAGITTAL}$	Sagittal	64×64	300, 300, 10	9.24	5.13	FLASH	16^	None
	$HR_{brain-3}^{FLASH-FS-SAGITTAL}$	Sagittal	256×256	300, 300, 10	15.97	9.00	FLASH	32	None
	$LR_{brain-3}^{FLASH-FS-AXIAL}$	Axial	64×64	300, 300, 10	9.24	5.13	FLASH	16^	None
	$HR_{brain-3}^{FLASH-FS-AXIAL}$	Axial	256×256	300, 300, 10	15.96	9.00	FLASH	32	None
	$LR_{brain-3}^{FLASH-FS-CORONAL}$	Coronal	64×64	300, 300, 10	9.24	5.13	FLASH	16^	None
	$HR_{brain-3}^{FLASH-FS-CORONAL}$	Coronal	256×256	300, 300, 10	15.96	9.00	FLASH	32	None
Phantom	$LR_{phantom}^{FLASH-FS}$	Axial	64×64×40	300, 300, 200	500	4.41	FLASH	4	None
	$HR_{phantom}^{FLASH-FS}$	Axial	256×256×40	300, 300, 200	500	4.41	FLASH	4	None
Thorax-1	$LR_{thorax-1}^{FLASH-FS}$	Coronal	64×64	400, 400, 10	9.10	5.18	FLASH	1 [per cine frame]	None
	$HR_{thorax-1}^{FLASH-FS}$	Coronal	256×256	400, 400, 10	13.86	7.79	FLASH	1 [per cine frame]	None
Thorax-2	$LR_{thorax-2}^{SSFB-FS}$	Coronal	64×64	400, 400, 10	3.79	1.87	SSFB	1 [per cine frame]	None
	$LR_{thorax-2}^{SSFB-US}$	Coronal	64×64	400, 400, 10	3.79	1.87	SSFB	1 [per cine frame]	2×
Thorax-3	$LR_{thorax-3}^{FLASH-US}$	Coronal	64×64	400, 400, 10	2.90	3.55	FLASH	1 [per cine frame]	2×
	$HR_{thorax-3}^{FLASH-US}$	Coronal	256×256	400, 400, 10	5.11	7.05	FLASH	1 [per cine frame]	2×
Latency	$LR_{latency}^{FLASH-US}$	Sagittal	64×64	400, 400, 10	2.90	3.55	FLASH	1 [per cine frame]	2×
	$HR_{latency}^{FLASH-US}$	Sagittal	256×256	400, 400, 10	5.11	7.05	FLASH	1 [per cine frame]	2×

Supplementary table 2: Quantitative results of super-resolution on a NIST phantom. Each up-sample method (nearest, bicubic interpolation, $EDSR_{brain}$, and $EDSR_{thorax}$) were compared directly to their reference image. Numbers reported are the arithmetic mean across the 25 slices. NRMSE: normalised root mean-square-error, SSIM: structural similarity, EDSR: enhanced deep super-resolution.

**Up-sample method**	**NRMSE**	**SSIM**
Nearest	5.9%	0.870
Bicubic	4.7%	0.889
$EDSR_{brain}$	4.5%	0.892
$EDSR_{thorax}$	4.5%	0.892
