# Supplementary material for: Super-resolution neural networks improve the spatiotemporal resolution of adaptive MRI-guided radiation therapy
Source: Commun Med (Lond). 2024 Apr 4;4:64. doi: 10.1038/s43856-024-00489-9 (PMC10994938; doi:10.1038/s43856-024-00489-9)
Supplement: Supplementary file 2 — Description of Additional Supplementary Files [file 43856_2024_489_MOESM2_ESM.pdf]

Description of Additional Supplementary Files

File name: Supplementary Data 1
Description: Spreadsheet of the data used to generate boxplots is stored as ‘supplementary data 1’. Each sheet of the spreadsheet corresponds to the figure (or subfigure).

File name: Supplementary Data 2
Description: Zip file containing short GIFs of cine-MRI with/without super-resolution. This is referred to in the text as ‘supplementary data 2’. The filename for each GIF corresponds to the acquisition as detailed in the supplementary table 2
