# Supplementary figures and images for: Super-resolution neural networks improve the spatiotemporal resolution of adaptive MRI-guided radiation therapy
Source: Commun Med (Lond). 2024 Apr 4;4:64. doi: 10.1038/s43856-024-00489-9 (PMC10994938; doi:10.1038/s43856-024-00489-9)

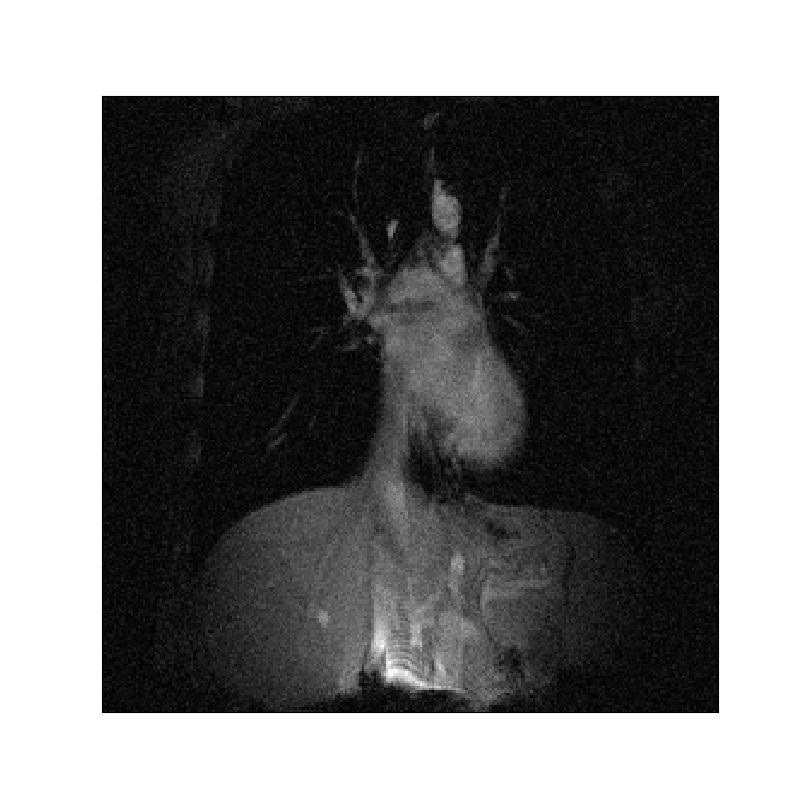

Supplement: Supplementary file 4 — Supplementary Data 2 [file 43856_2024_489_MOESM4_ESM.zip › Supplementary data 2/Thorax-1/HR_thorax-1^FLASH-FS.gif]

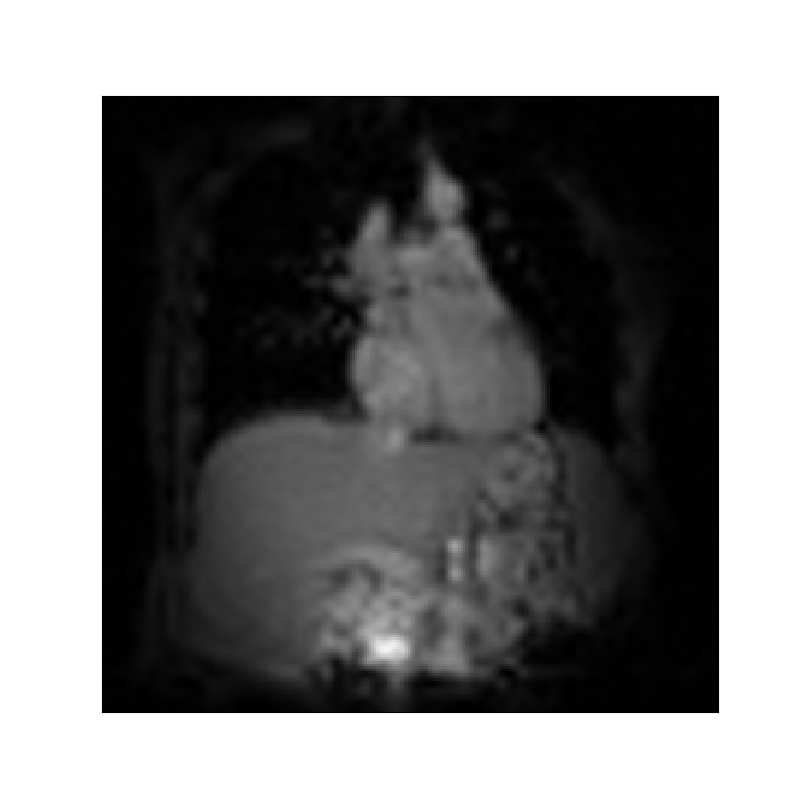

Supplement: Supplementary file 4 — Supplementary Data 2 [file 43856_2024_489_MOESM4_ESM.zip › Supplementary data 2/Thorax-1/LR_thorax-1^FLASH-FS with bicubic interpolation.gif]

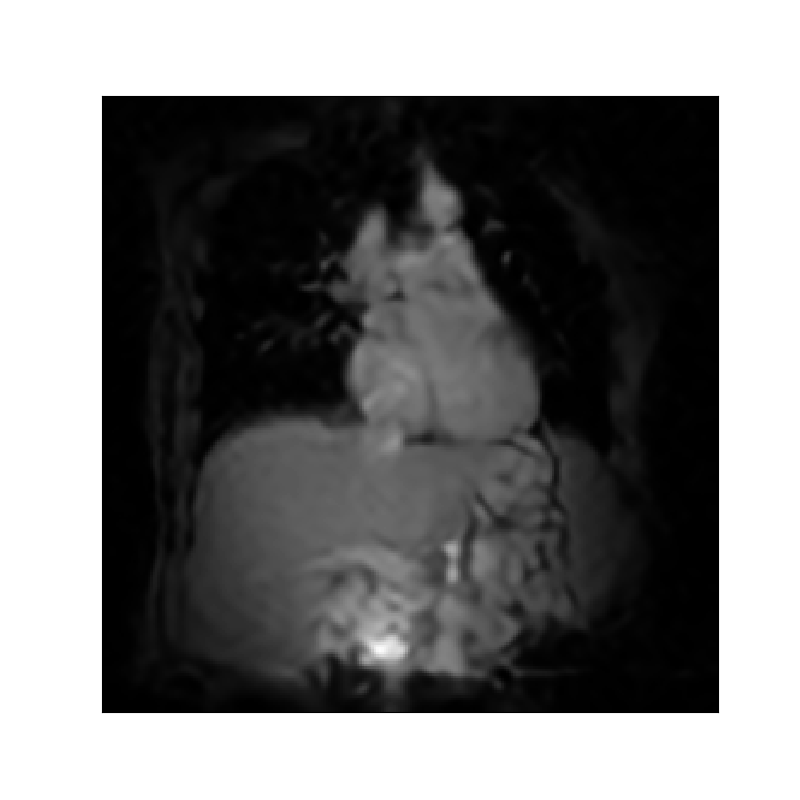

Supplement: Supplementary file 4 — Supplementary Data 2 [file 43856_2024_489_MOESM4_ESM.zip › Supplementary data 2/Thorax-1/LR_thorax-1^FLASH-FS with EDSRthorax.gif]

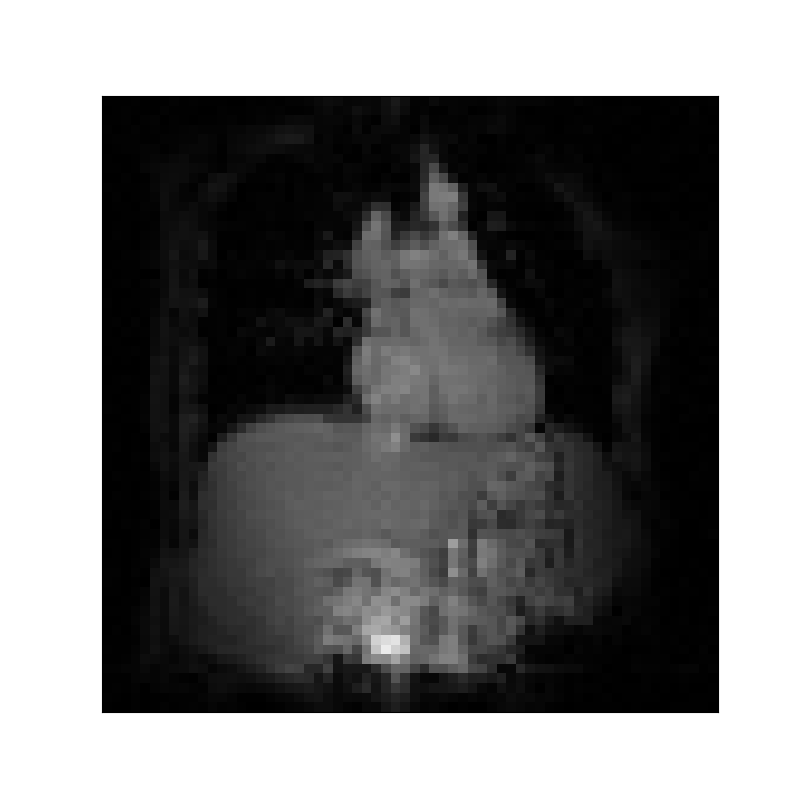

Supplement: Supplementary file 4 — Supplementary Data 2 [file 43856_2024_489_MOESM4_ESM.zip › Supplementary data 2/Thorax-1/LR_thorax-1^FLASH-FS.gif]

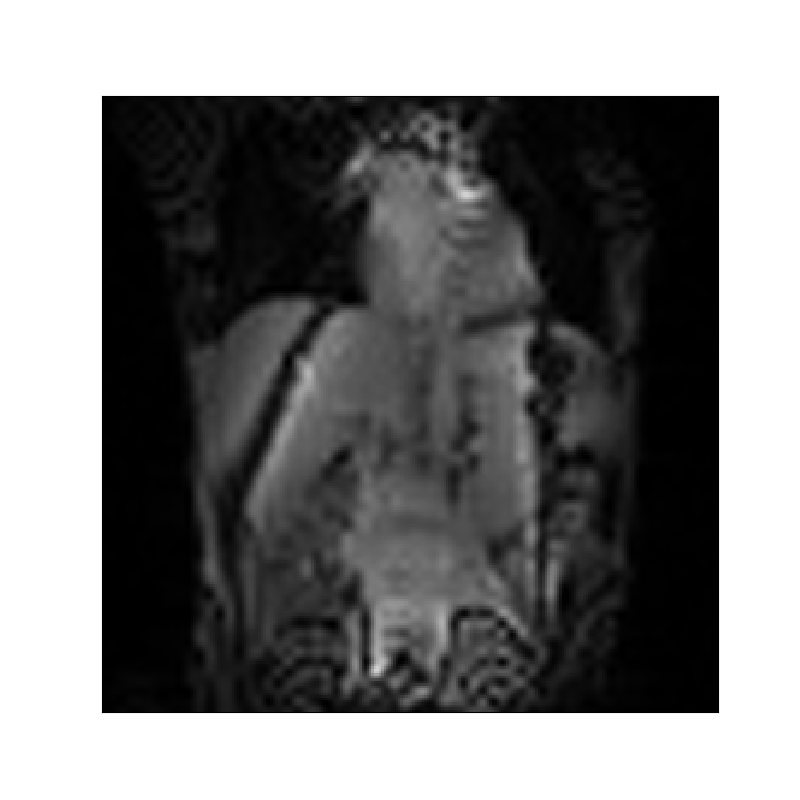

Supplement: Supplementary file 4 — Supplementary Data 2 [file 43856_2024_489_MOESM4_ESM.zip › Supplementary data 2/Thorax-2/LR_thorax-2^SSFB-FS with bicubic interpolation.gif]

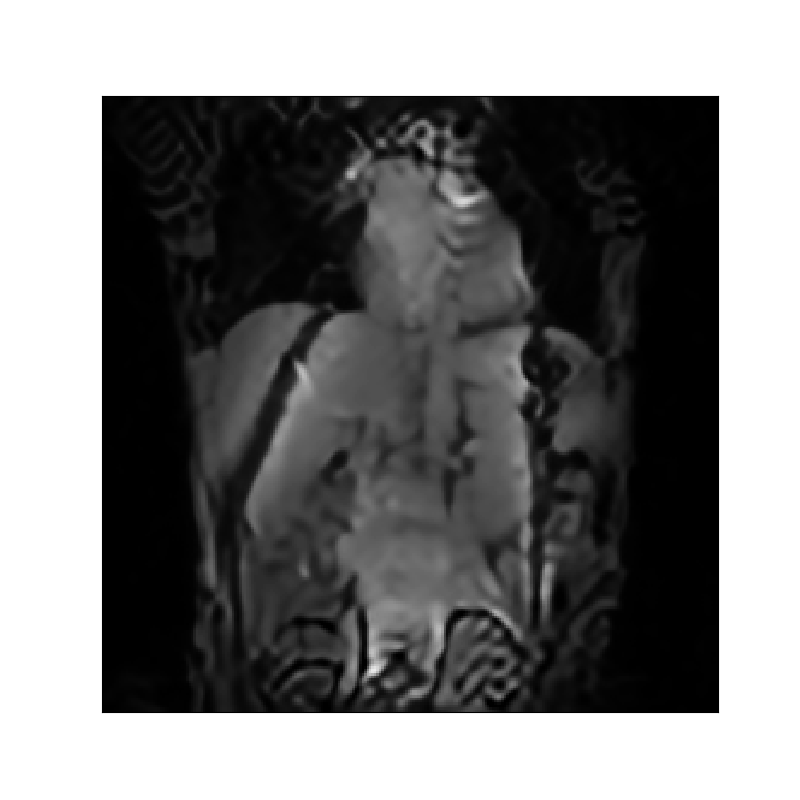

Supplement: Supplementary file 4 — Supplementary Data 2 [file 43856_2024_489_MOESM4_ESM.zip › Supplementary data 2/Thorax-2/LR_thorax-2^SSFB-FS with EDSRthorax.gif]

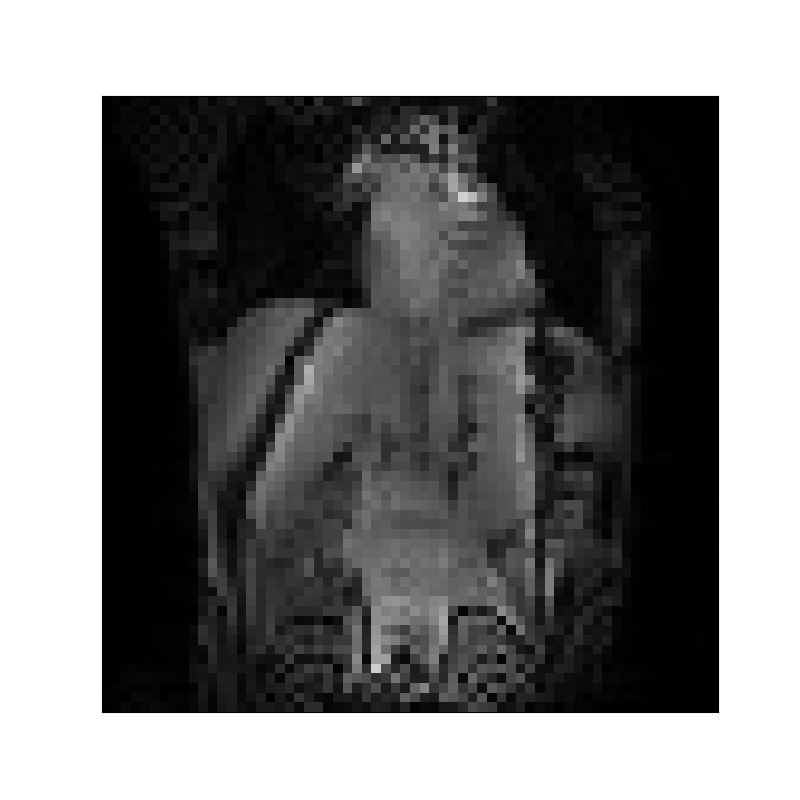

Supplement: Supplementary file 4 — Supplementary Data 2 [file 43856_2024_489_MOESM4_ESM.zip › Supplementary data 2/Thorax-2/LR_thorax-2^SSFB-FS.gif]

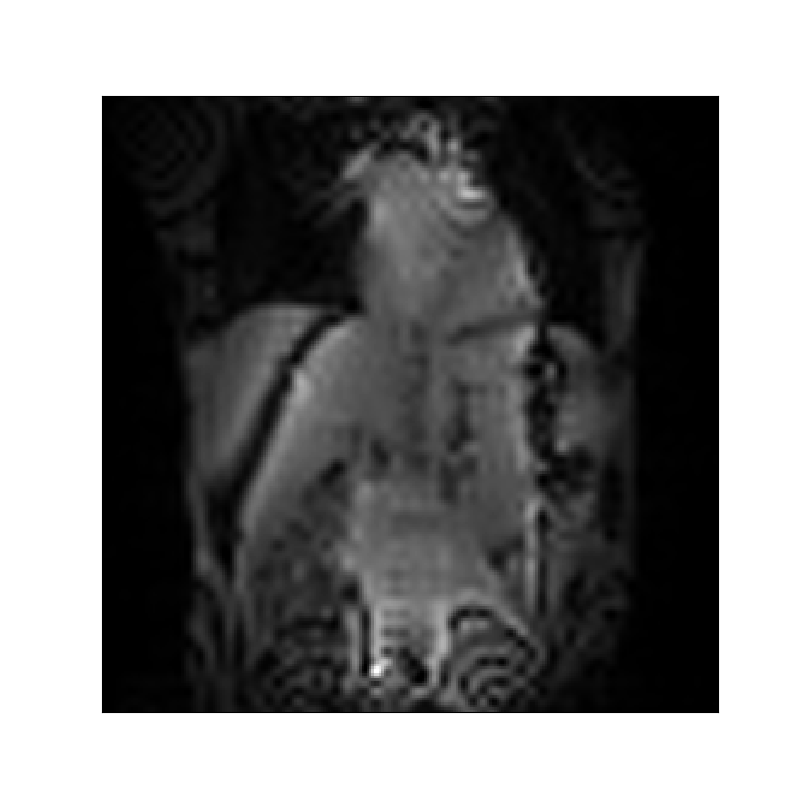

Supplement: Supplementary file 4 — Supplementary Data 2 [file 43856_2024_489_MOESM4_ESM.zip › Supplementary data 2/Thorax-2/LR_thorax-2^SSFB-US with bicubic interpolation.gif]

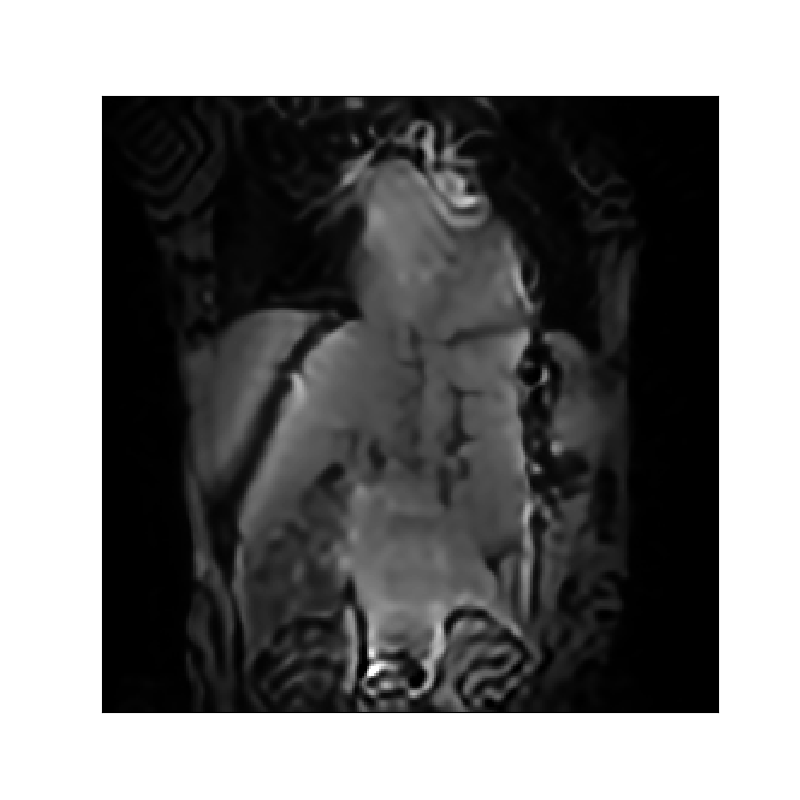

Supplement: Supplementary file 4 — Supplementary Data 2 [file 43856_2024_489_MOESM4_ESM.zip › Supplementary data 2/Thorax-2/LR_thorax-2^SSFB-US with EDSRthorax.gif]

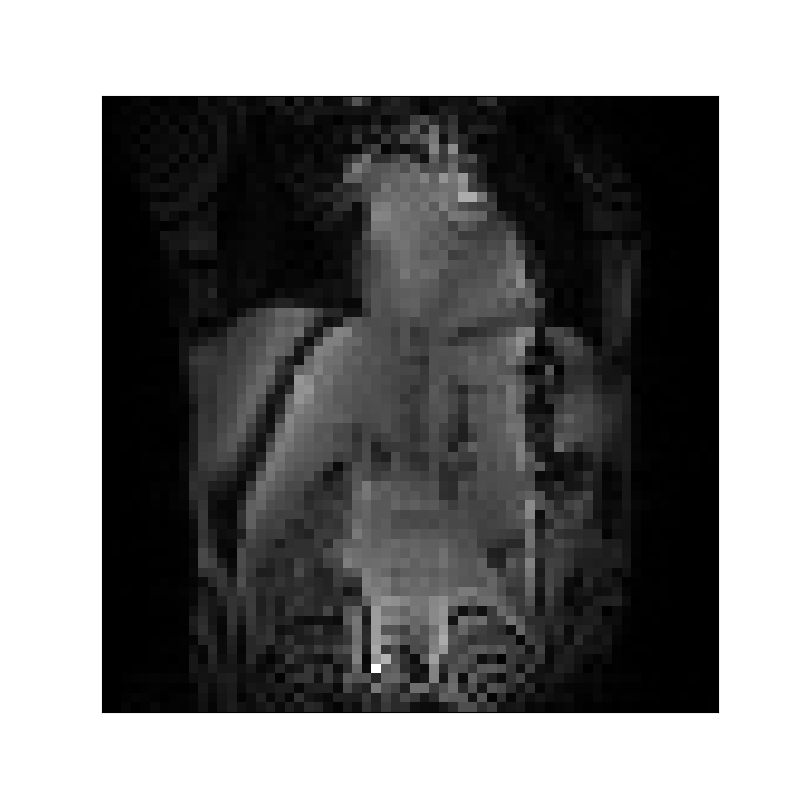

Supplement: Supplementary file 4 — Supplementary Data 2 [file 43856_2024_489_MOESM4_ESM.zip › Supplementary data 2/Thorax-2/LR_thorax-2^SSFB-US.gif]

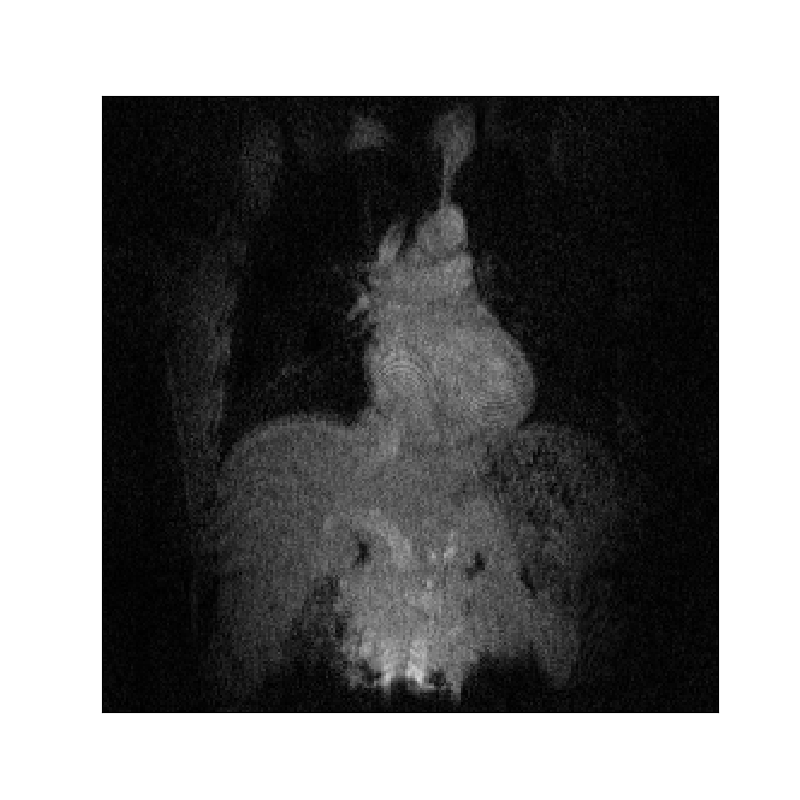

Supplement: Supplementary file 4 — Supplementary Data 2 [file 43856_2024_489_MOESM4_ESM.zip › Supplementary data 2/Thorax-3/HR_thorax-3^FLASH-US.gif]

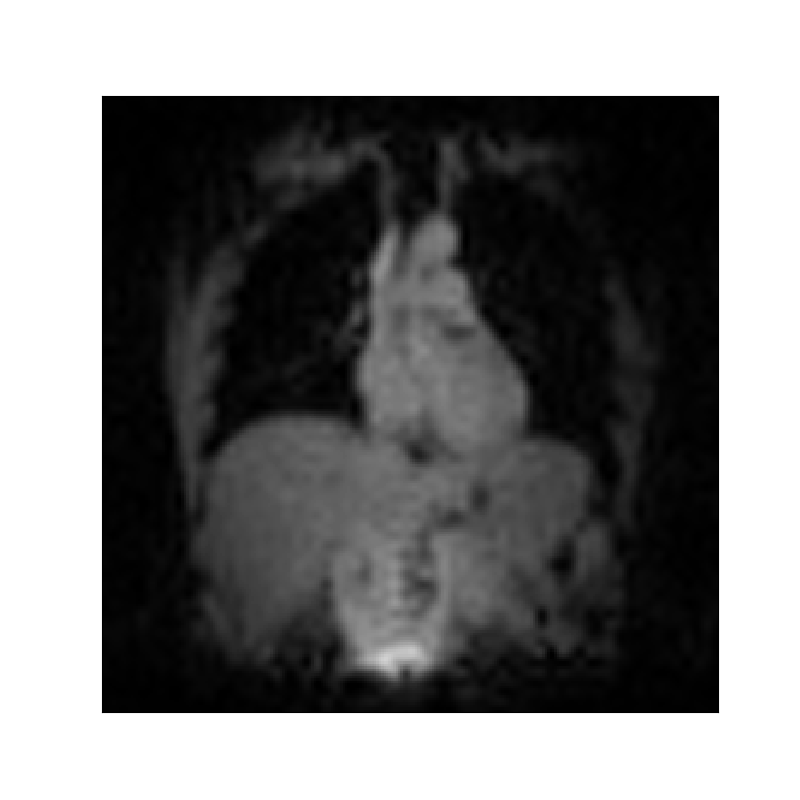

Supplement: Supplementary file 4 — Supplementary Data 2 [file 43856_2024_489_MOESM4_ESM.zip › Supplementary data 2/Thorax-3/LR_thorax-3^FLASH-US with bicubic interpolation.gif]

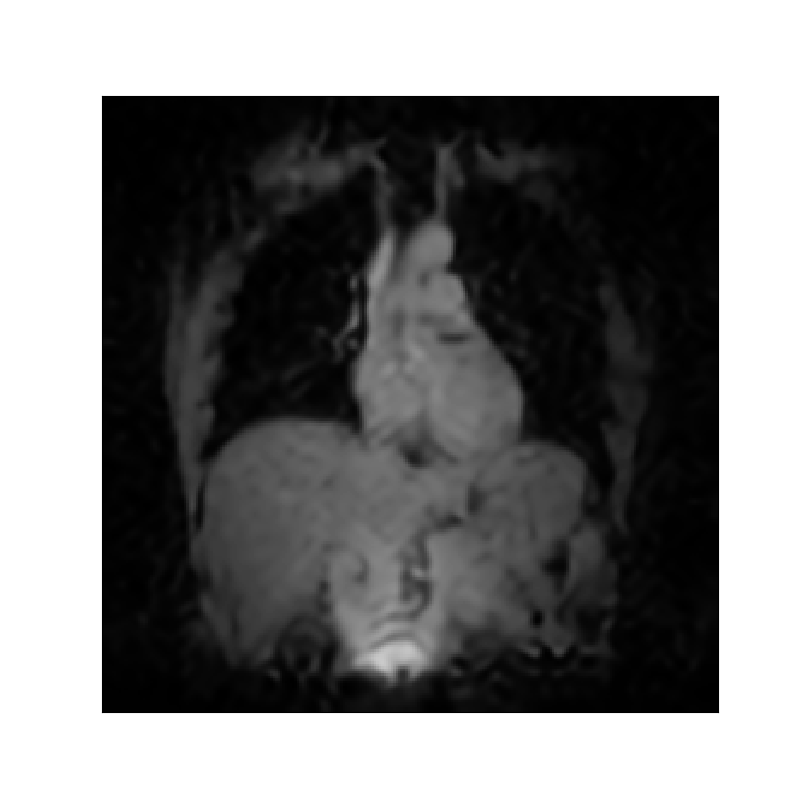

Supplement: Supplementary file 4 — Supplementary Data 2 [file 43856_2024_489_MOESM4_ESM.zip › Supplementary data 2/Thorax-3/LR_thorax-3^FLASH-US with EDSRthorax.gif]

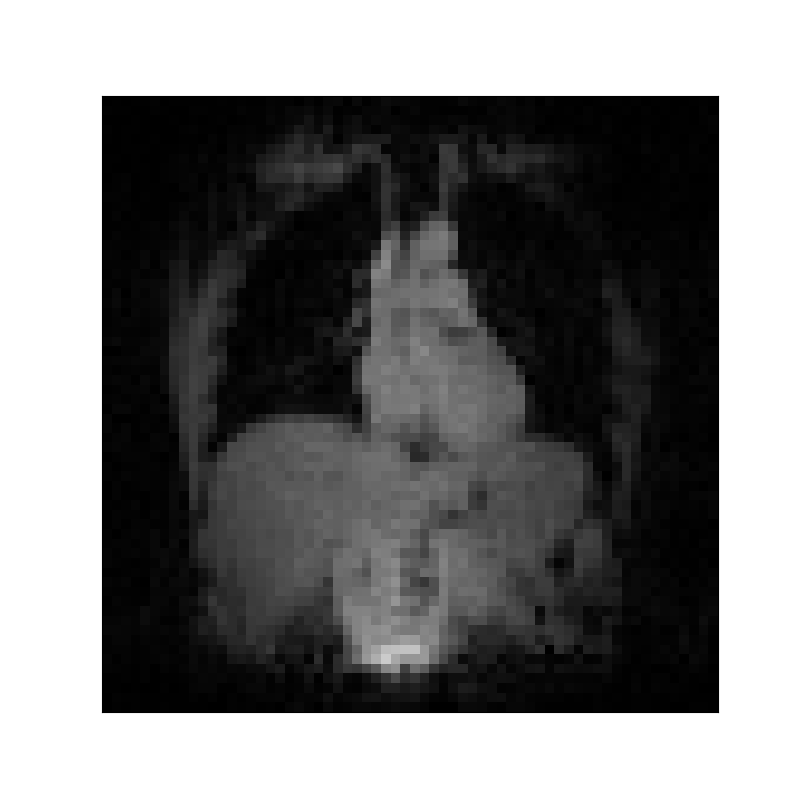

Supplement: Supplementary file 4 — Supplementary Data 2 [file 43856_2024_489_MOESM4_ESM.zip › Supplementary data 2/Thorax-3/LR_thorax-3^FLASH-US.gif]
