# Supplementary material for: Super-resolution neural networks improve the spatiotemporal resolution of adaptive MRI-guided radiation therapy
Source: Commun Med (Lond). 2024 Apr 4;4:64. doi: 10.1038/s43856-024-00489-9 (PMC10994938; doi:10.1038/s43856-024-00489-9)
Supplement: Supplementary file 5 — Reporting Summary [file 43856_2024_489_MOESM5_ESM.pdf]

nature portfolio

Corresponding author(s): James Grover

Last updated by author(s): 22nd March 2024

Reporting Summary

Nature Portfolio wishes to improve the reproducibility of the work that we publish. This form provides structure for consistency and transparency in reporting. For further information on Nature Portfolio policies, see our Editorial Policies and the Editorial Policy Checklist.

Statistics

For all statistical analyses, confirm that the following items are present in the figure legend, table legend, main text, or Methods section.

n/a	Confirmed	
☐	☒	The exact sample size (n) for each experimental group/condition, given as a discrete number and unit of measurement
☐	☒	A statement on whether measurements were taken from distinct samples or whether the same sample was measured repeatedly
☐	☒	The statistical test(s) used AND whether they are one- or two-sided<br>*Only common tests should be described solely by name; describe more complex techniques in the Methods section.*
☒	☐	A description of all covariates tested
☐	☒	A description of any assumptions or corrections, such as tests of normality and adjustment for multiple comparisons
☐	☒	A full description of the statistical parameters including central tendency (e.g. means) or other basic estimates (e.g. regression coefficient) AND variation (e.g. standard deviation) or associated estimates of uncertainty (e.g. confidence intervals)
☐	☒	For null hypothesis testing, the test statistic (e.g. F, t, r) with confidence intervals, effect sizes, degrees of freedom and P value noted<br>*Give P values as exact values whenever suitable.*
☒	☐	For Bayesian analysis, information on the choice of priors and Markov chain Monte Carlo settings
☒	☐	For hierarchical and complex designs, identification of the appropriate level for tests and full reporting of outcomes
☒	☐	Estimates of effect sizes (e.g. Cohen's d, Pearson's r), indicating how they were calculated

Our web collection on statistics for biologists contains articles on many of the points above.

Software and code

Policy information about availability of computer code

Data collection	Integration code available at https://github.com/Image-X-Institute/MRI-Linac_SuperResolution
Data analysis	Image quality metrics were performed using Python (v. 3.9) the scikit-image package (v. 0.16.2). Tracking latency and accuracy was performed using MATLAB 2019a. Anatomical figures were generated using the matplotlib package (v. 3.5.1).

For manuscripts utilizing custom algorithms or software that are central to the research but not yet described in published literature, software must be made available to editors and reviewers. We strongly encourage code deposition in a community repository (e.g. GitHub). See the Nature Portfolio guidelines for submitting code & software for further information.

Data

Policy information about availability of data

All manuscripts must include a data availability statement. This statement should provide the following information, where applicable:

- Accession codes, unique identifiers, or web links for publicly available datasets
- A description of any restrictions on data availability
- For clinical datasets or third party data, please ensure that the statement adheres to our policy

QIN-GBM Treatment Response (https://wiki.cancerimagingarchive.net/display/Public/QIN+GBM+Treatment+Response), UPENN-GBM (https://wiki.cancerimagingarchive.net/pages/viewpage.action?pageId=70225642), and Prostate-Diagnosis (https://wiki.cancerimagingarchive.net/display/Public/PROSTATE-DIAGNOSIS) are available on the Cancer Imaging Archive for approved research use upon agreement with their respective terms of use and

license31,33,42,43. AVATAR and MRI-linac volunteer data cannot be shared publicly at this time. The terms of patient/volunteer consent for these studies allow for de-identified data to be shared with external research institutions under a formal collaboration agreement. Researchers wishing to access these data should contact the corresponding author.
The data points plotted in figures 2, 4 and supplementary figure 2 can be found in supplementary data 1.

Research involving human participants, their data, or biological material

Policy information about studies with human participants or human data. See also policy information about sex, gender (identity/presentation), and sexual orientation and race, ethnicity and racism.

Reporting on sex and gender	Populations characteristics (including sex and gender) were not considered when sourcing healthy volunteer participants.
Reporting on race, ethnicity, or other socially relevant groupings	No socially constructed or socially relevant categorization variables were used in this manuscript. Populations characteristics (including race, ethnicity, and other socially relevant groupings) were not considered when sourcing healthy volunteer participants.
Population characteristics	See above.
Recruitment	Healthy volunteers were recruited under the 'Magnetic Resonance Imaging in healthy volunteers' non-interventional study at Liverpool hospital.
Ethics oversight	South Western Sydney Local Health District Human Research Ethics Committee

Note that full information on the approval of the study protocol must also be provided in the manuscript.

Field-specific reporting

Please select the one below that is the best fit for your research. If you are not sure, read the appropriate sections before making your selection.

☒ Life sciences ☐ Behavioural & social sciences ☐ Ecological, evolutionary & environmental sciences

For a reference copy of the document with all sections, see nature.com/documents/nr-reporting-summary-flat.pdf

Life sciences study design

All studies must disclose on these points even when the disclosure is negative.

Sample size	In the case of publicly available datasets, 630 (for UPENN-GBM) and 89 (for prostate-diagnosis) patients were used. Image quality metrics were performed on three healthy volunteers with similar imaging acquisitions producing a dataset of 9 orthogonal slices for MRI-linac data. Tracking latency and accuracy measurements were repeated 3 times.
Data exclusions	No data were excluded from the analysis.
Replication	Three healthy volunteers were imaged. Tracking latency and geometric accuracy experiments were repeated three times.
Randomization	The entirety of the publicly available datasets (UPENN-GBM and prostate-diagnosis) were used for performance characterisation. Randomisation was not relevant for the healthy volunteer imaging as all were used in the quantitative analysis.
Blinding	Blinding was not relevant to this imaging study. All participants were imaged using the same methodology. Quantitative analysis of the results were automated to ensure reproducibility.

Reporting for specific materials, systems and methods

We require information from authors about some types of materials, experimental systems and methods used in many studies. Here, indicate whether each material, system or method listed is relevant to your study. If you are not sure if a list item applies to your research, read the appropriate section before selecting a response.

Materials & experimental systems

n/a	Involved in the study
☒	☐ Antibodies
☒	☐ Eukaryotic cell lines
☒	☐ Palaeontology and archaeology
☒	☐ Animals and other organisms
☒	☐ Clinical data
☒	☐ Dual use research of concern
☒	☐ Plants

Methods

n/a	Involved in the study
☒	☐ ChIP-seq
☒	☐ Flow cytometry
☐	☒ MRI-based neuroimaging

Magnetic resonance imaging

Experimental design

Design type: Image quality comparison, this was not an fMRI study.

Design specifications: N/A

Behavioral performance measures: N/A

Acquisition

Imaging type(s): Structural

Field strength: 1.0 T

Sequence & imaging parameters: See supplementary information 3 for all imaging parameters.

Area of acquisition: Whole brain.

Diffusion MRI: ☐ Used ☒ Not used

Preprocessing

Preprocessing software: SimpleITK was used to register the MRIs.

Normalization: Images were clipped to their 99.9th percentile to remove extremely bright spots. Image were subsequently min-max normalised between 0 and their maximum to equalise the noise floor.

Normalization template: N/A

Noise and artifact removal: N/A

Volume censoring: N/A

Statistical modeling & inference

Model type and settings: N/A

Effect(s) tested: N/A

Specify type of analysis: ☒ Whole brain ☐ ROI-based ☐ Both

Statistic type for inference (See Eklund et al. 2016): N/A

Correction: N/A

Models & analysis

n/a	Involved in the study
☒	☐ Functional and/or effective connectivity
☒	☐ Graph analysis
☒	☐ Multivariate modeling or predictive analysis
